# Inflammasome activation negatively regulates MyD88-IRF7 type I IFN signaling and anti-malaria immunity

**DOI:** 10.1038/s41467-018-07384-7

**Published:** 2018-11-23

**Authors:** Xiao Yu, Yang Du, Chunmei Cai, Baowei Cai, Motao Zhu, Changsheng Xing, Peng Tan, Meng Lin, Jian Wu, Jian Li, Mingjun Wang, Helen Y. Wang, Xin-zhuan Su, Rong-Fu Wang

**Affiliations:** 1 Center for Inflammation and Epigenetics, Houston Methodist Research Institute, Houston, TX 77030 USA; 2 Department of Immunology, School of Basic Medical Sciences, Southern Medical University, 510515 Guangzhou, Guangdong People’s Republic of China; 3 Zhongshan School of Medicine, Sun Yat-sen University, 510275 Guangzhou, Guangdong People’s Republic of China; 4 Institute of Biosciences and Technology, College of Medicine, Texas A & M University, Houston, TX 77030 USA; 5 School of Life Sciences, Sun Yat-sen University, 510275 Guangzhou, Guangdong People’s Republic of China; 6 Laboratory of Malaria and Vector Research, National Institute of Allergy and Infectious Diseases, National Institutes of Health, Bethesda, MD 20892 USA; 7 State Key Laboratory of Cellular Stress Biology, Innovation Center for Cell Biology, School of Life Sciences, Xiamen University, 361005 Xiamen, Fujian People’s Republic of China; 8 Department of Microbiology and Immunology, Weill Cornell Medicine, Cornell University, New York, NY 10065 USA

## Abstract

The inflammasome plays a critical role in inflammation and immune responses against pathogens. However, whether or how inflammasome activation regulates type I interferon (IFN-I) signaling in the context of malaria infection remain unknown. Here we show mice deficient in inflammasome sensors AIM2, NLRP3 or adaptor Caspase-1 produce high levels of IFN-I cytokines and are resistant to lethal *Plasmodium yoelii* YM infection. Inactivation of inflammasome signaling reduces interleukin (IL)-1β production, but increases IFN-I production. Mechanistically, we show inflammsome activation enhances IL-1β-mediated MyD88-TRAF3-IRF3 signaling and SOCS1 upregulation. However, SOCS1 inhibits MyD88-IRF7-mediated-IFN-I signaling and cytokine production in plasmacytoid dendritic cells. By contrast, ablation of inflammsome components reduces SOCS1 induction, and relieves its inhibition on MyD88-IRF7-dependent-IFN-I signaling, leading to high levels of IFN-α/β production and host survival. Our study identifies a previously unrecognized role of inflammasome activation in the negative regulation of IFN-I signaling pathways and provides potential targets for developing effective malaria vaccines.

## Abstract (web summary)

The inflammasome is an essential component of inflammatory processes and the host response to infection. Here the authors show that inflammasome activation modulates MyD88-IRF7 type I IFN signalling and anti-malaria immunity.

## Introduction

Malaria is a deadly infectious disease that affects ~225 million people, resulting in about 348,000 deaths in 2015^1^. One of the hallmarks of malaria blood stage is high cyclical fevers and elevated levels of inflammatory mediators^2,3^. During malaria infection, *Plasmodium*-specific pathogen-associated molecular patterns (PAMPs), including glycosylphosphatidylinositol (GPI), hemozoin, genomic DNA (gDNA), and RNA, are recognized by host immune system, leading to activation of various signaling pathways such as type I interferon, nuclear factor κB (NF-κB) and inflammasome responses^4^. These signaling pathways have key roles in disease control and are tightly controlled. However, how these signaling pathways are regulated in vivo, particularly in response to malaria infection, remains poorly understood.

Inflammasome activation is a multiple-step processing for caspase-1 activation and production of mature IL-1β and IL-18 in response to infectious microbes^5,6^. Inflammasome signaling starts with recognition of PAMPs by pattern recognition receptors (PRRs), such as AIM2 (absent in melanoma 2), NLRP3 (NLR family pyrin domain containing 3) or NLRC4 (NLR family card domain containing 4), leading to formation of inflammasomes, activation of caspase-1, and cleavage of pro-IL-1β and pro-IL-18 to generate IL-1β and IL-18^5,7^. Additionally, some gram-negative bacteria and fungal infections can also activate non-canonical inflammasome pathways that activate caspase-11^8^ or caspase-8^9^. IL-1β has an important role in controlling infections caused by *Salmonella typhimurium* and *Candida albicans*^10–12^; however, excessive amount of IL-1β is also associated with auto-inflammatory diseases^13^. Recent studies show that malaria hemozoin can activate the NLRP3 inflammasome in experimental cerebral malaria^14,15^. Furthermore, infected erythrocytes can activate both NLRP3 and AIM2 inflammasomes during malaria in vitro stimulation^16^. Despite these important studies, it remains unknown whether inflammasome activation pathway has a beneficial or detrimental effect on host immunity and mortality during lethal *Plasmodium yoelii* YM infection.

Type I IFN signaling has an important role in malaria infection^17^. Activations of cGAS/STING, MDA5/MAVS, TLR7-MyD88-IRF7 signaling pathways have been reported in response to malaria infections through recognition of parasite DNA and RNA^18–22^. In particular, we previously showed that mice deficient in MAVS/STING-mediated IRF3-dependent type I IFN signaling produce higher levels of type I IFN cytokines and were resistant to lethal YM infection^19^. Importantly, cGAS/STING-mediated and MDA5/MAVS-mediated IRF3-dependent signaling inhibits MyD88-IRF7-mediated type I IFN response in wild-type (WT) plasmacytoid dendritic cells (pDCs) through upregulation of SOCS1, leading to lethal phenotype in WT mice^19^. Several studies have shown that type I IFN regulates inflammasome activity through several distinct mechanisms^23–28^. However, whether and how inflammasome activation regulates type I IFN signaling pathways and antimalarial immunity is poorly understood.

In this study, we sought to define the role of inflammasome activation in lethal *P. yoelii* YM infection, and the potential mechanisms that inflammasome signaling regulates type I IFN signaling and anti-malaria immunity in vivo. We showed that mice deficient in genes of inflammasome signaling (*Aim2*^−/−^, *Nlrp3*^−/−^, *Casp1*^−/−^, and *Il1r1*^−/−^) were resistant to lethal *P. yoelii* YM infection. We further demonstrated that inflammasome activation and IL-1 signaling regulated type I IFN signaling and SOCS1 expression through a MyD88-TRAF3-IRF3-dependent pathway, which inhibits MyD88-dependent IRF7-mediated type I IFN signaling pathway in pDCs. Our findings have identified a previously unrecognized regulatory mechanism of inflammasome signaling in type I IFN response against *P. yoelii* YM infection, and provided potential therapeutic targets for the development of effective malaria vaccines.

## Results

### Inflammasome-deficient mice are resistant to YM infection

**Fig. 1:**
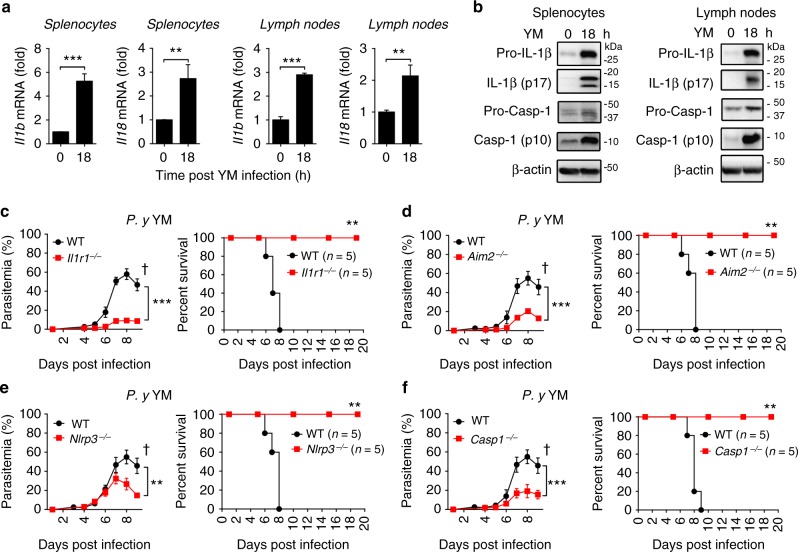
Inflammasome deficiency augments the resistance to lethal *P. yoelii* YM infection in vivo. **a**, **b** WT (*n* = 5) mice were intraperitoneally infected with *P. yoelii* YM (0.5 × 10^6^ iRBCs); spleen and lymph nodes were collected at indicated times post infection and subjected to qPCR (**a**) and immunoblotting analyses (**b**). **c**–**f** WT and *Il1r1*^*−/−*^ (**c**), *Aim2*^−/−^ (**d**), *Nlrp3*^−/−^ (**e**), *Casp1*^−/−^ (**f**) mice (*n* = 5) were intraperitoneally infected with *P. yoelii* YM (0.5 × 10^6^ iRBCs). Daily parasitemias and mortality rates are monitored. Data are representative of three independent experiments and plotted as mean ± SD. **p < 0.01, ***p < 0.001 vs. corresponding control. Dagger denotes mouse death

Previous studies show that AIM2 and NLRP3 function as sensors of malaria gDNA and hemozoin to trigger inflammasome activation in vitro^16^. However, the in vivo role of these sensors (AIM2, NLRP3) and their downstream signaling molecules in lethal malaria infection remains to be defined. To address this issue, we first evaluated inflammasome response during lethal malaria infection in vivo by intraperitoneally injection of wild-type (WT) C57BL/6 mice with lethal *P. yoelii* YM (YM for short) infected red blood cells (iRBC). We found that IL-1β and IL-18 mRNA, as well as protein levels of mature IL-1β (p17) and cleaved fragment of pro-caspase-1 (p10) were significantly increased at 18 h post infection (p.i.) (Fig. 1a, b), suggesting that inflammasome signaling was activated at early stage of YM infection. Since IL-1R is required for IL-1β produced by inflammasome activation, we next infected 6–8 weeks-old WT and *Il1r1*^−/−^ mice with a lethal dose (0.5 × 10^6^ iRBCs) of YM, and monitored parasitemia and disease progression. The infected *Il1r1*^−/−^ mice had significantly lower parasitemia from day 5 than those of infected WT mice, and were still alive at 30 days p.i., but WT mice died by day 9 (Fig. 1c and Supplementary Fig. 1a). These results suggest that inflammasome activity has a detrimental role during YM infection. Next, we investigated which of the inflammasome sensors and downstream signaling molecules are involved during lethal YM infection. We infected *Aim2*^−/−^, *Nlrp3*^−/−^, and *Casp1*^−/−^ mice with YM-iRBCs, and found that parasitemias in *Aim2*^−/−^, *Nlrp3*^−/−^, and *Casp1*^−/−^ mice were markedly lower than those in WT mice. Importantly, these knockout (KO) mice were completely resistant to lethal YM infection (Fig. 1d–f and Supplementary Fig. 1b–d). These results suggest that activation of AIM2-mediated and NLRP3-mediated Caspase-1-dependent inflammasome signaling negatively regulates host immune responses against lethal YM infection.

### AIM2 and NLRP3 are required for inflammasome activation

**Fig. 2:**
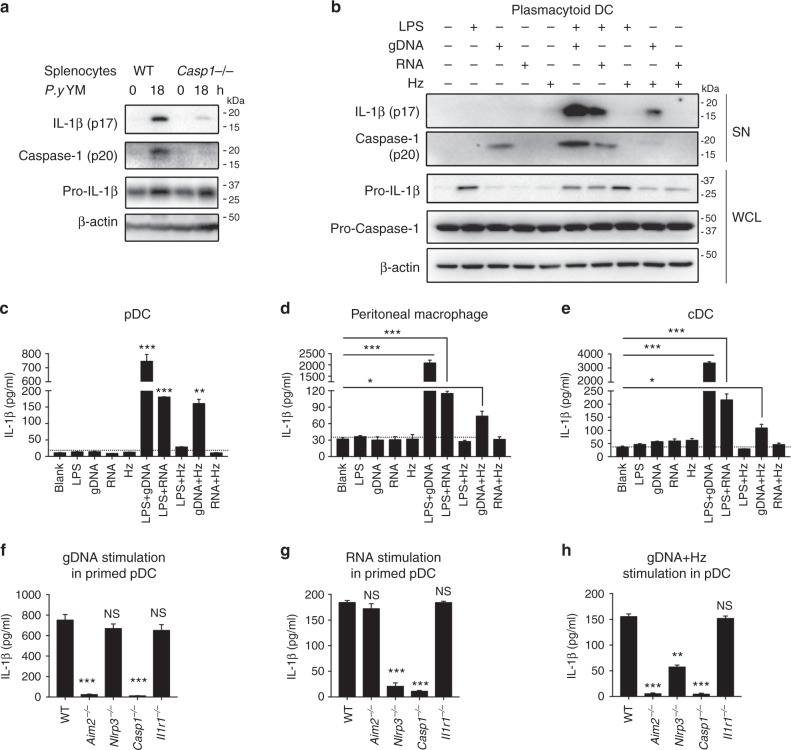
*Plasmodium* gDNA activates innate immune AIM2 inflammasome, while RNA and hemozoin trigger NLRP3 inflammasome activation. **a** WT and *Casp1*^−/−^ mice (*n* = 5) were infected with *P. yoelii* YM, and splenocytes were harvested at 18 h after infection. Uninfected samples served as control group. Cell lysates were analyzed by immunoblotting with the indicated antibodies. **b**, **c** WT pDCs were stimulated as indicated for 24 h, cell lysate and supernatants were collected for immunoblotting analysis (**b**), and supernatants were collected for quantization of IL-1β cytokine by ELISA analysis (**c**). **d**, **e** WT peritoneal macrophages (**d**) and cDCs (**e**) were stimulated as indicated for 24 h, and supernatants were collected for quantization of IL-1β cytokine by ELISA analysis. **f**, **g** LPS-primed WT, *Il1r1*^−/−^, *Aim2*^−/−^, *Nlrp3*^−/−^ and *Casp1*^−/−^ pDCs were stimulated with gDNA (**f**) or RNA (**g**) for 24 h, and supernatants were collected for quantization of IL-1β cytokine by ELISA analysis. **h** WT, *Il1r1*^−/−^, *Aim2*^−/−^, *Nlrp3*^−/−^, and *Casp1*^−/−^ pDCs were stimulated with gDNA/hemozoin complex for 24 h, supernatants were collected for quantization of IL-1β cytokine by ELISA analysis. Data are representatives of three independent experiments with similar results and plotted as mean ± SD. *p < 0.05, **p < 0.01 vs. corresponding control. NS, not significant

To understand the activation of inflammasome signaling in WT and *Casp1*^−/−^ mice in vivo, we examined inflammasome activation and cytokine expression after YM infection, and found marked decrease in cleavages of IL-1β and Caspase-1 in *Casp1*^−/−^ splenocytes at 18 h p.i. (Fig. 2a). To further identify immune cell population responsible for inflammasome activation and cytokine production, we stimulated plasmacytoid dendritic cells (pDCs), conventional dendritic cells (cDCs) and peritoneal macrophages from WT mice with gDNA, RNA or/and hemozoin purified from YM. We found that YM gDNA or RNA alone could not induce the production of IL-1β, but could trigger mature IL-1β production in LPS-primed pDCs, cDCs and peritoneal macrophages (Fig. 2b–e and Supplementary Fig. 2a, b). The combination of gDNA and Hz could also stimulate IL-1β production. As expected, deficiency in *Aim2* or *Casp1* markedly reduced gDNA triggered IL-1β maturation in pDCs, cDCs and macrophages (Fig. 2f and Supplementary Fig. 2c–l), whereas the deficiency of *Nlrp3* significantly affected RNA triggered IL-1β maturation (Fig. 2g and Supplementary Fig. 2m, n), indicating that gDNA activation of inflammasome depends on AIM2, while RNA recognition requires NLRP3. We also found that mature IL-1β production after hemozoin/gDNA complex stimulation was decreased in *Nlrp3*^−/−^ pDCs, and was totally abolished in *Aim2*^−/−^ and *Casp1*^−/−^ pDCs (Fig. 2h), which is consistent with previous findings, showing that hemozoin carries gDNA into lysosomal compartment and activates NLRP3 and AIM2 inflammasome^16^. Taken together, these results suggest that malaria gDNA, RNA and hemozoin activate inflammasome through AIM2 and NLRP3 signaling during YM infection.

### IL-1β activates IFN-I signaling through MyD88-TRAF3-IRF3

**Fig. 3:**
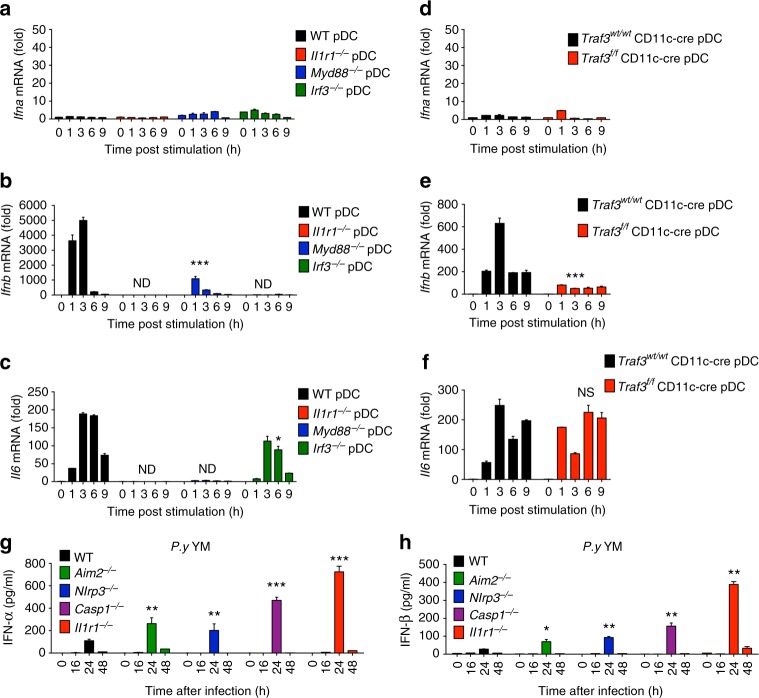
IL-1 signaling activates MyD88-TRAF3-IRF3-dependent type I IFN signaling in vitro and negatively regulates type I IFN cytokine production in vivo. **a**–**c** WT, *Il1r1*^−/−^, *Myd88*^−/−^, and *Irf3*^−/−^ pDCs were stimulated with recombinant mouse IL-1β for indicated times, and RNA from pDCs was isolated and used for expression analysis of *Ifna* (**a**)*, Ifnb* (**b**), and *Il6* (**c**) by using qPCR. **d**–**f** *Traf3*^*wt/wt*^ *CD11c-cre* and *Traf3*^*f/f*^ *CD11c-cre* pDCs were stimulated with recombinant mouse IL-1β for indicated times, and RNA from cells was isolated and used for expression analysis of *Ifna* (**d**)*, Ifnb* (**e**), and *Il6* (**f**) by using qPCR. **g**, **h** WT, *Aim2*^−/−^, *Nlrp3*^−/−^, *Casp1*^−/−^, and *Il1r1*^−/−^ mice were intraperitoneally infected with *P. yoelii* YM. Serum was collected at indicated times and subjected to ELISA analysis of IFN-α (**g**) and IFN-β (**h**). Data are representatives of three independent experiments with similar results and plotted as mean ± SD. *p < 0.05, **p < 0.01, ***p < 0.001 vs. corresponding control. NS, not significant, ND, not detected

Since our in vivo studies showed that inflammasome activation negatively regulates host immunity against lethal YM infection, we next sought to determine molecular mechanisms and downstream signaling induced by recombinant mouse IL-1β cytokine, and found that IL-1β stimulation activates IFN-β and IL-6, but not IFN-α mRNAs in WT pDCs (Fig. 3a–c), cDCs (Supplementary Fig. 3a) and BMDM (Supplementary Fig. 3b). *Ifnb* expression was abolished in *Il1r1*^−/−^, *Myd88*^−/−^, and *Irf3*^−/−^ pDCs (Fig. 3b), suggesting that IL-1β induced *Ifnb* expression is dependent on IL1R1-Myd88-IRF3 axis. Expression of *Il6* was only abolished in *Il1r1*^−/−^ and *Myd88*^−/−^ pDCs. Similar results were obtained in WT, *Il1r1*^−/−^ and *Myd88*^−/−^ cDCs and BMDM (Supplementary Fig. 3a, b). To determine whether TRAF3 is required for *Ifnb* expression, we generated *Traf3*^*f/f*^ CD11c-cre mice by crossing *Traf3*^*f/f*^ mice with CD11c-cre mice, and isolated pDCs and cDCs from WT and *Traf3*^*f/f*^ CD11c-cre mice. After IL-1β stimulation, we found that expression of *Ifnb*, but not IL-6, was diminished in *Traf3*^*f/f*^ CD11c-cre pDCs and cDCs, compared with *Traf3*^*wt/wt*^ CD11c-cre pDCs (Fig. 3d–f) and cDCs (Supplementary Fig. 3c), suggesting that TRAF3 is essential for IL-1β induced type I IFN signaling and *Ifnb* expression.

### Inflammasome activation inhibits IFN-I cytokine production

Although our study showed that IL-1β-treated immune cells produce type I IFN cytokines through the MyD88-TRAF3-IRF3 pathway, how inflammasome triggers type I IFN production in vivo is still elusive. For this reason, we infected WT, *Aim2*^−/−^, *Nlrp3*^−/−^, *Casp1*^−/−^, and *Il1r1*^−/−^ mice with YM iRBCs and examined the serum levels of IFN-α/β, IL-6, and IFN-γ. In sharp contrast to in vitro data, we unexpectedly found that the serum amounts of IFN-α and IFN-β in *Aim2*^−/−^, *Nlrp3*^−/−^, *Casp1*^−/−^, and *Il1r1*^−/−^ mice were much higher than those in WT mice at 24 h after YM infection (Fig. 3g, h and Supplementary Fig. 3d–h), suggesting that AIM2, NLRP3, CASP1, or IL1R1 deficiency enhances type I IFN cytokine production in vivo after YM infection.

### MyD88 is required for robust production of IFN-α and IFN-β

**Fig. 4:**
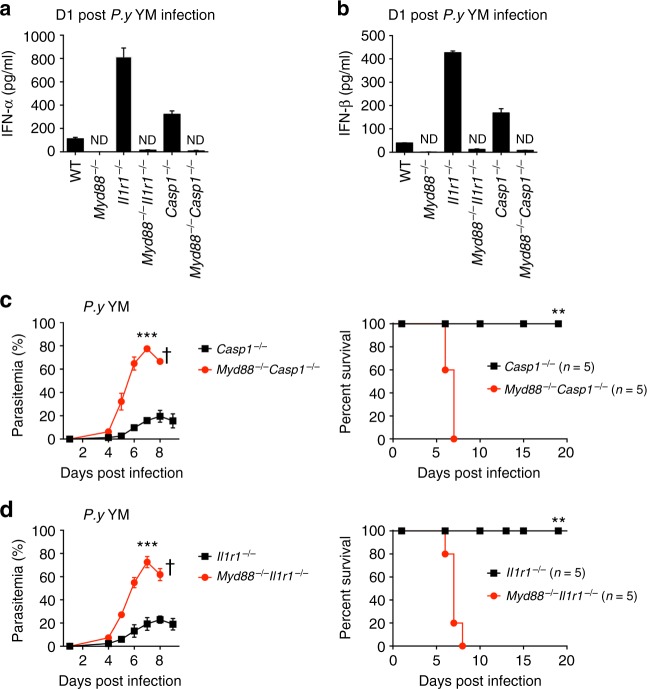
Activation of inflammasome negatively regulates type I IFN cytokine production in a MyD88-dependent manner. WT, *Myd88*^−/−^, *Il1r1*^−/−^, *Casp1*^−/−^, *Myd88*^−/−^*Il1r1*^−/−^, and *Myd88*^−/−^*Casp1*^−/−^ mice (*n* = 5) were intraperitoneally infected with *P. yoelii* YM. Serum was collected at 24 h post infection and subjected to ELISA analysis of IFN-α (**a**) and IFN-β (**b**). Parasitemias and survivals (**c** and **d**) were monitored daily. Data are representatives of three independent experiments with similar results and plotted as mean ± SD. **p < 0.01, ***p < 0.001 vs. corresponding control. ND, not detected. Dagger denotes mouse death

We next investigated how inflammasome activation affects type I IFN response during lethal YM infection. We crossed *Il1r1*^−/−^ and *Casp1*^−/−^ mice with *Myd88*^−/−^ mice to generate double knockout (DKO) mice, and then infected these single and double KO mice with lethal YM. We found that enhanced IFN-α and IFN-β levels were completely abolished in DKO mice (Fig. 4a, b), suggesting that the enhanced IFN-α and IFN-β production in *Il1r1*^−/−^ and *Casp1*^−/−^ mice is MyD88-dependent. Consistently, parasitemia and mortality in DKO mice were markedly increased, compared with those of *Il1r1*^−/−^ or *Casp1*^−/−^ mice (Fig. 4c, d). Taken together, these results suggest that MyD88 is essential for the enhanced type I IFN cytokine production in inflammasome-deficient mice.

### pDCs are the major cells for IFN-α and IFN-β production

**Fig. 5:**
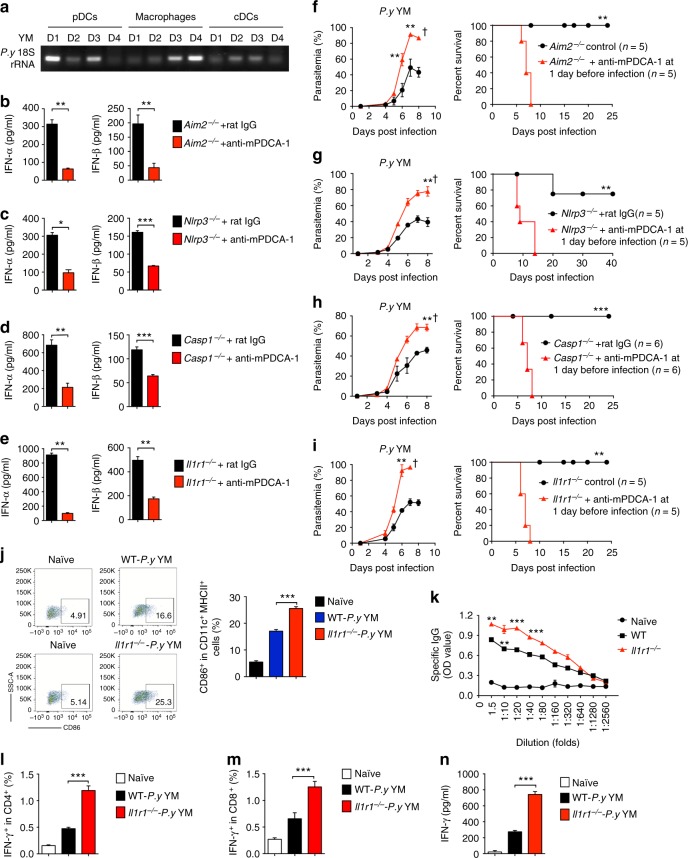
pDCs contribute to the production of type I IFN at the early time post *P. yoelii* YM infection and ameliorate pathogenesis in inflammasome-deficient mice. **a** The cell populations of pDCs, cDCs, and macrophages were isolated from WT mice splenocytes at indicated times post YM infection using cell isolation kits, and then analyzed for cell-specific expression of *P. yoelii* 18 S rRNA by PCR. **b**–**e** *Aim2*^−/−^ (**b**), *Nlrp3*^−/−^ (**c**), *Casp1*^−/−^ (**d**), and *Il1r1*^−/−^ (**e**) mice (*n* = 5) were injected with anti-mPDCA-1 antibody at 12 h before and after infection, followed by *P. yoelii* YM infection. Serum was collected at 24 h post infection and subjected to ELISA analysis for IFN-α and IFN-β. **f**–**i** *Aim2*^−/−^ (**f**), *Nlrp3*^−/−^ (**g**), *Casp1*^−/−^ (**h**), and *Il1r1*^−/−^ (**i**) mice (*n* = 5) were injected with anti-mPDCA-1 antibody at 12 h before and after infection, followed by *P. yoelii* YM infection. Parasitemias and survivals were monitored daily. **j** WT and *Il1r1*^−/−^ mice (*n* = 5) were infected with YM. Splenocytes were collected at day 4 post infection, and CD86^+^ cells in CD11c^+^MHC-II^+^ cells were measured by flow cytometry. Representative FACS and statistical analysis of CD86-positive cells are shown. **k** Malaria specific IgG in serum from WT and *Il1r1*^−/−^ mice (*n* = 5) at day 14 post YM infection, evaluated using ELISA.  **l**–**n** Intracellular staining of IFN-γ were measured by flow cytometry in splenocytes of WT and *Il1r1*^−/−^ mice (*n* = 5) at day 10 post YM infection, followed by stimulation with crude antigens (YM iRBCs). Statistical analysis is shown in **l** and **m**. Splenocytes from YM-infected WT and *Il1r1*^−/−^ mice were cultured with crude antigens (iRBCs) overnight, and cell supernatants were collected for ELISA analysis (**n**).  Data are representative of three independent experiments and plotted as mean ± SD. *p < 0.05, **p < 0.01, ***p < 0.001 vs. corresponding control. Dagger denotes mouse death

Because IL-1β produced by inflammasome signaling pathway during malaria infection can trigger type I IFN signing in pDCs, cDCs and macrophages through IL-1R-mediated MyD88-TRAF3-IRF3 signaling pathway, we next sought to identify the major cell population responsible for early IFN-α and IFN-β production in vivo in inflammasome-deficient mice. We first isolated pDCs, cDCs and macrophages from WT mice at different times post YM infection, and found that malaria 18S rRNA was only detected in pDCs, but not in cDCs and macrophages at 18 h post YM infection (Fig. 5a), suggesting that pDC is mainly responsible for uptake of YM parasite nucleic acid during infection. To further demonstrate the direct involvement of pDCs for early IFN-α and IFN-β production, we depleted pDCs in *Aim2*^−/−^, *Nlrp3*^−/−^, *Casp1*^−/−^, and *Il1r1*^−/−^ mice by injection of anti-mPDCA-1 antibody at 12 h before and 12 h after infection. We found that mPDCA-1-mediated depletion of pDCs in *Aim2*^−/−^, *Nlrp3*^−/−^, *Casp1*^−/−^, and *Il1r1*^−/−^ mice markedly decreased serum levels of IFN-α/β at day 1 after YM infection, compared with those treated with control antibody (Fig. 5b–e). Consistent with these findings, pDC depletion by mPDCA-1 antibody also significantly increased parasitemias and mortality in *Aim2*^−/−^, *Nlrp3*^−/−^, *Casp1*^−/−^, and *Il1r1*^−/−^ mice infected with lethal YM (Fig. 5f–i). These results suggest that pDCs are the major cell population responsible for early IFN-α and IFN-β production and resistance to lethal YM infection in inflammasome-deficient mice.

To exclude the possibility that macrophages and cDCs also contribute to early IFN-α and IFN-β production, we also depleted macrophages in *Nlrp3*^−/−^ or *Aim2*^−/−^ mice by intraperitoneal injection of clodronate liposome at 2 days before YM infection. We showed no significant changes in serum cytokine levels (IFN-α/β) at 24 h *p.i*., parasitemias and mortality rates (Supplementary Fig. 4a–c). It should be noted that macrophages could produce IFN-β, but not IFN-α, after IL-1β stimulation (Supplementary Fig. 3b), but the amounts of IFN-β in macrophages were small, compared with that in pDCs. In addition, macrophages were not the major cell population for uptake of malaria nucleic acid. Together, these factors may explain why we did not detect the difference in IFN-β production after depletion of macrophages in *Nlrp3*^−/−^ and *Aim2*^−/−^ mice. However, we found that macrophage depletion in *Nlrp3*^−/−^ and *Aim2*^−/−^ mice at 1 day after YM infection markedly increased parasitemia and host mortality (Supplementary Fig. 4d, e), suggesting a critical role of macrophages during the later stage YM infection. We also generated *Il1r1*^−/−^ cDC-DTR mice to deplete cDC population upon diphtheria toxin (DT) administration, and found that cDC depletion at 4 days before YM infection did not change parasitemia and survival (Supplementary Fig. 4f), suggesting that cDCs are not responsible for early stage anti-malaria immunity. However, depletion of cDCs at 1 day after YM infection converted resistant *Il1r1*^−/−^ mice to sensitive to YM infection (Supplementary Fig. 4f). Taken together, these results suggest that macrophages and cDCs are not required for early IFN-α/β cytokine production (within the first 24 h of infection), but have indispensable roles at the late stage (after 24 h of infection) of YM infection in *Nlrp3*^−/−^ and *Aim2*^−/−^ mice.

### Robust IFN-I production enhances adaptive immune responses

Next, we sought to determine how inflammasome-deficient mice generate better immune responses against YM infection. To determine whether robust production of type I IFN in inflammasome-deficient mice contributed to dendritic cell maturation, we infected WT and *Il1r1*^−/−^ mice with YM and then isolated splenocytes at day 4 post infection for mature DC population analysis. We found that the percentages of CD86^+^ cells in MHCII^+^CD11c^+^ cells from *Il1r1*^−/−^ mice were much higher than those cells from WT mice (Fig. 5j and Supplementary Fig. 5a), suggesting that YM-infected *Il1r1*^−/−^ mice promote dendritic cell maturation. To examine whether inflammasome-deficient mice develop better B cell responses, we infected WT and *Il1r1*^−/−^ mice with 0.1 × 10^6^ YM iRBCs (five times lower dosage to keep WT mice survive longer) and collected serum at day 14 post infection. We found that the concentrations of malaria specific IgG immunoglobulin in serum were significantly higher in *Il1r1*^−/−^ mice than in WT mice (Fig. 5k). To further demonstrate whether inflammasome-deficient mice generate better T cell responses, we isolated splenocytes at day 6 post infection and stimulated with crude antigens (*P.y* YM-iRBC) for T cell function analysis. Our results showed that the percentage of splenic IFN-γ^+^ cells in CD4^+^ and CD8^+^ cells from *Il1r1*^−/−^ mice were much higher than those cells from WT mice (Fig. 5l, m and Supplementary Fig. 5b, c). Consistently, IFN-γ protein levels in the supernatant of splenocytes after overnight culture with crude antigens were significantly higher in *Il1r1*^−/−^ mice than in WT mice (Fig. 5n). Taken together, these results suggest that YM-infected *Il1r1*^−/−^ mice developed potent adaptive immune responses and higher quality of B and T cell responses than WT mice.

### Induction of SOCS1 by IL-1β inhibits IFN-I signaling

**Fig. 6:**
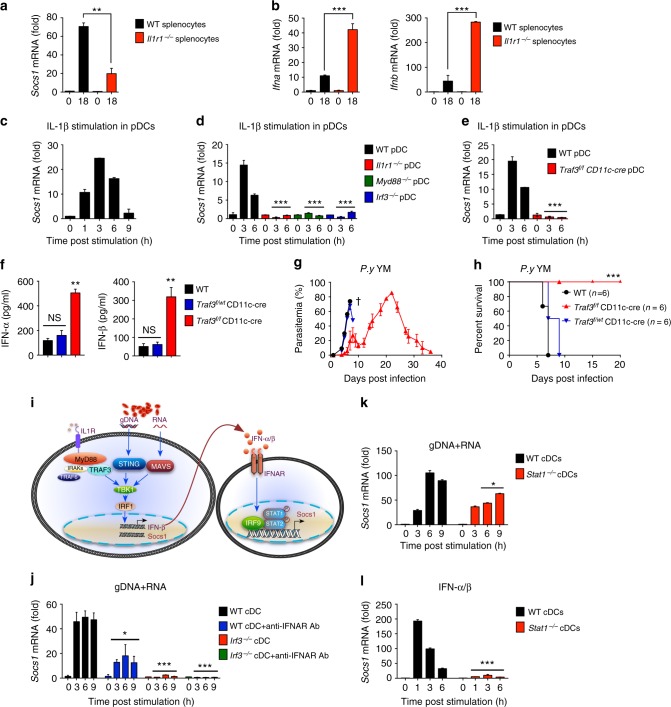
Negative regulator SOCS1 is directly induced by IL-1β signaling in a MyD88-TRAF3-IRF3-dependent type I IFN signaling. **a**, **b** WT and *Il1r1*^−/−^ mice (*n* = 5) were infected with *P. yoelii* YM for indicated times, and RNA from splenocytes was isolated and used for expression analysis of *Socs1* (**a**), *Ifna* and *Ifnb* (**b**) by using qPCR. **c** WT pDCs were stimulated with recombinant mouse IL-1β (2 μg/ml) for indicated times, RNA from the pDCs was isolated and used for expression analysis of *Socs1* by using qPCR. **d** WT, *Il1r1*^−/−^, *Myd88*^−/−^, and *Irf3*^−/−^ pDCs were stimulated with recombinant IL-1β (2 μg/ml) for indicated times, RNA from pDCs was isolated and used for expression analysis of *Socs1* by using qPCR. **e** WT and *Traf3*^*f/f*^ *CD11c-cre* pDCs were stimulated with recombinant IL-1β (2 μg/ml) for indicated times, RNA from pDCs was isolated and used for expression analysis of *Socs1* by using qPCR. **f**–**h** WT, *Traf3*^*f/f*^ *CD11c-cre*, and *Traf3*^*f/wt*^ *CD11c-cre* mice (*n* = 5) were infected with *P. yoelii* YM. Serum was collected at 24 h post infection and subjected to ELISA analysis for IFN-α, IFN-β (**f**). Parasitemias (**g**) and survivals (**h**) were monitored daily. **i** A model to show whether SOCS1 is directly induced by IRF3-dependent signaling or the downstream IFNAR-mediated signaling. **j** WT and *Irf3*^−/−^ cDCs were pretreated with or without anti-IFNAR antibody for 24 h and stimulated with YM gDNA plus RNA for indicated times. RNA from cDCs was isolated and used for expression analysis of *Socs1* by using qPCR. **k**, **l** WT and *Stat1*^−/−^ cDCs were stimulated with YM gDNA plus RNA (**k**) or mouse IFN-α/β (**l**) for indicated times. RNA from splenocytes was isolated and used for expression analysis of *Socs1* by using qPCR. Data are representative of three independent experiments and plotted as mean ± SD. *p < 0.05, **p < 0.01, ***p < 0.001 vs. corresponding control. NS, not significant. Dagger denotes mouse death

To further elucidate the mechanisms by which ablation of inflammasome signaling enhances MyD88-IRF7 dependent type I IFN production in pDCs, we examined the expression pattern of several potential negative regulators in freshly isolated splenocytes from YM-infected WT and *Il1r1*^−/−^ mice, and found that the mRNA of *Socs1* were markedly increased in WT, but little in IL1R1-deficient splenocytes (Fig. 6a). By contrast, we did not observe appreciable changes in the expression of *Socs2*, *Socs3*, *Pcbp2*, *Rnf5, Inpp5d*, *Inppl1*, *Otud5*, *Nlrc3*, and *Ube3c* between YM-infected WT and *Il1r1*^−/−^ mice (Supplementary Fig. 6a). Importantly, *Socs1* mRNA abundance was inversely correlated with *Ifna* and *Ifnb* mRNA in splenocytes from YM-infected WT and *Il1r1*^−/−^ mice (Fig. 6b). Similar results were obtained from WT and *Nlrp3*^−/−^ mice (Supplementary Fig. 6b). Collectively, these findings indicate that SOCS1 can be induced by inflammasome-coupled IL-1 signaling after YM infection, and negatively regulates MyD88-IRF7 type I IFN signaling.

To further confirm these observations, we purified WT pDCs and stimulated with recombinant IL-1β, and found that SOCS1 was directly induced by IL-1β in a time-dependent manner (Fig. 6c). The IL-1β stimulated SOCS1 expression was abolished in pDCs isolated from *Il1r1*^−/−^, *Myd88*^−/−^, and *Irf3*^−/−^ mice (Fig. 6d), suggesting that IL-1β-induced SOCS1 expression is dependent on IL-1R-Myd88-IRF3 axis. Furthermore, we showed that expression of SOCS1 was also diminished in pDCs isolated from *Traf3*^*f/f*^ CD11c-cre mice compared with WT pDCs (Fig. 6e), suggesting that TRAF3 is essential for the production of SOCS1 upon IL-1β stimulation. Consistently, serum levels of IFN-α and IFN-β in *Traf3*^*f/f*^ CD11c-cre mice were much higher than in WT and *Traf3*^*f/wt*^ CD11c-cre mice 24 h after YM infection (Fig. 6f), leading to significantly reduce parasitemia and host mortality (Fig. 6g, h). As expected, SOCS1 expression was reduced in cDCs and BMDM isolated from *Il1r1*^−/−^, *Myd88*^−/−^, *Irf3*^−/−^ and *Traf3*^*f/f*^ CD11c-cre mice (Supplementary Fig. 6c–e), but there was no MyD88-IRF7 type I IFN signaling in these cells.

Because *SOCS1* is an ISG gene (induced by IFN-α/β) and inhibits downstream signaling of IFN-α/β by inhibiting JAK1^29,30^, we asked whether SOCS1 could be induced by two distinct pathways: IRF3-mediated signaling or IFN-α/β-IFN receptor-mediated downstream signaling (Fig. 6i). To address this issue, we stimulated WT and *Irf3*^−/−^ cDCs with malaria gDNA and RNA, in the absence or presence of anti-IFNAR antibody, and found that SOCS1 induction was markedly reduced in WT cDCs in the presence of anti-IFNAR antibody, and completely abolished in *Irf3*^−/−^ cDCs regardless of anti-IFNAR antibody treatment (Fig. 6j). Furthermore, we showed that SOCS1 expressions were reduced in *Stat1*^−/−^ cDCs and splenocytes treated with malaria gDNA and RNA, but completely abolished in *Stat1*^−/−^ cells with IFN-α/β stimulation (Fig. 6k, l and Supplementary Fig. 6f, g). These results suggest that SOCS1 can be induced by IRF3-dependent type I IFN signaling and its downstream IFN receptor-mediated signaling.

### Fine-tuned regulation of SOCS1 dictates resistance

**Fig. 7:**
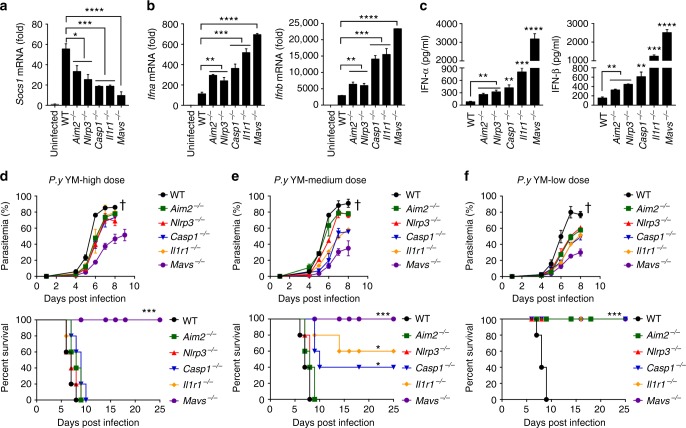
Fine-tuned regulation of SOCS1 induction by inflammasome and type I IFN signaling dictates host resistance to YM infection. **a**–**c** WT and deficient (*Aim2*^−/−^, *Nlrp3*^−/−^, *Casp1*^−/−^, *Il1r1*^−/−^, and *Mavs*^−/−^) mice (*n* = 5) were infected with YM, RNA from pDCs was isolated at 18 h post infection and used for expression analysis of *Socs1* (**a**), *Ifna* and *Ifnb* (**b**) by using qPCR, and serum levels of IFN-α and IFN-β in WT and deficient mice at 24 h after *P. yoelii* YM infection are shown in **c**. **d**–**f** WT and deficient (*Aim2*^−/−^, *Nlrp3*^−/−^, *Casp1*^−/−^, *Il1r1*^−/−^, and *Mavs*^−/−^) mice (*n* = 5) were infected with high dose (1 × 10^6^ iRBCs, **d**), medium dose (0.75 × 10^6^ iRBCs, **e**) or low dose (0.5 × 10^6^ iRBCs, **f**) of *P. yoelii* YM. Daily parasitemias and mortality rates of WT and deficient mice after *P. yoelii* YM infection are shown. Data are plotted as mean ± SD and representative of three independent experiments with similar results. *p < 0.05, **p < 0.01, ***p < 0.001 vs. corresponding control. NS, not significant. Dagger denotes mouse death

Our previous study suggests that DNA sensor/adaptor (cGAS-STING)- and RNA sensor/adaptor (MDA5-MAVS)-induced signaling pathways converge at TBK1 to activate IRF3-mediated SOCS1 expression^19^. This study shows that inflammasome-coupled IL-1 signaling can also induce SOCS1 expression via the IL-1R-MyD88-TRAF3-IRF3 axis. A key question is how blocking one of these pathways can markedly affect SOCS1 induction, which, in turn, inhibits MyD88-IRF7-mediated type I IFN signaling in pDCs. To address this issue, we isolated pDCs from YM-infected WT and gene deficient (*Aim2*^−/−^, *Nlrp3*^−/−^, *Casp1*^−/−^, *Il1r1*^−/−^, and *Mavs*^−/−^) mice at 18 h post YM infection and assessed mRNA expression levels of SOCS1, IFN-α and IFN-β. We found that expression was dramatically decreased in *Mavs*^−/−^ pDCs, modest decreased in *Casp1*^−/−^ and *Il1r1*^−/−^ pDCs, and slightly decreased in *Aim2*^−/−^ and *Nlrp3*^−/−^ pDCs, compared with WT pDCs (Fig. 7a). SOCS1 expression levels inversely correlated with levels of IFN-α and IFN-β mRNA expression (Fig. 7b). To further determine whether the inhibitory efficiency of MyD88-IRF7-mediated type I IFN signaling by inflammasome signaling- and MAVS-induced SOCS1 expression is correlated with protective immunity in vivo, we infected WT, *Aim2*^−/−^, *Nlrp3*^−/−^, *Casp1*^−/−^, *Il1r1*^−/−^, and *Mavs*^−/−^ mice with high dosage (1 × 10^6^ iRBCs, two times higher dosage than previously used), medium-dosage (0.75 × 10^6^ iRBCs) or low-dosage (0.5 × 10^6^ iRBCs) of YM. We found that IFN-α and IFN-β levels in various KO mice were significantly higher than that in WT mice, with *Mavs*^−/−^ mice having the highest cytokine levels (Fig. 7c). In particular, *Mavs*^−/−^ mice produced lowest level of SOCS1, highest level of IFN-α and IFN-β, and were completely resistant to YM infection, even with a high-dose. By contrast, all other KO mice were sensitive to high-dose YM infection (Fig. 7d). In medium-dose infection, *Casp1*^−/−^ and *Il1r1*^−/−^ mice were partially resistant (Fig. 7e). In low dose YM infection, all KO, but not WT, mice were resistant (Fig. 7f). These results suggest that MAVS deficiency confers the strongest inhibition of SOCS1 expression, results in the highest level of IFN-α and IFN-β production, and the strongest resistance to YM infection. By contrast, Casp1 and IL-1R deficiency is ranked in the middle, while AIM2 and NLRP3 deficiency is in the lowest group in resistance to YM infection.

## Discussion

Although AIM2 and NLRP3 have been identified as sensors of malaria gDNA and hemozoin in vitro, their in vivo function has not been reported. In this study, we show that mice deficient in AIM2, NLRP3, Caspase-1 or IL-1R1 are resistant to lethal YM infection. We further show that inflammasome activation by lethal YM infection negatively regulates MyD88-IRF7-dependent type I IFN signaling by IL-1β-mediated signaling pathway. Secreted IL-1β could activate IL-1R-mediated signaling in many cell types, including pDCs, macrophages and cDCs, to trigger MyD88-TRAF3-IRF3 signaling pathway for low level IFN-β production, but our results show that pDCs preferentially take up *Plasmodium* nucleic acid within the first 24 h post infection. This preferential pathogen nucleic acid uptake by pDCs leads to activation of two type I IFN signaling pathways: (1) IRF3-dependent type I IFN signaling pathway activated by IL-1β/IL-1R (this study), as well as cGAS/STING and MDA5/MAVS signaling; and (2) MyD88-IRF7-dependent pathway for production of large amounts of IFN-α/β production. Mechanistically, we show that AIM2 and NLRP3 inflammasome activation by YM infection produces IL-1β in pDCs and potentially many other cell types, depending upon exposure of malaria. IL-1β/IL-1R ligation triggers MyD88-TRAF3-IRF3-dependent type I IFN signaling for IFN-β expression and SOCS1 induction. Induced SOCS1 expression then inhibits MyD88-IRF7-dependent type I IFN signaling and IFN-α/β production in pDCs, but not in any other immune cells due to lack of expression of IRF7 (Supplementary Fig. 7a). Thus, our study links inflammasome pathway to activation and cross-regulation of two type I IFN signaling pathways, and illustrates fine-tuned regulation of SOCS1 induction, MyD88-IRF7 dependent type I IFN signaling and resistance to lethal malaria infection.

pDCs are the major sources of type I IFN at the early stage of YM infection^19,22^ and indispensable for host resistance. However, unlike cDCs and macrophages, activated pDCs cannot effectively present antigens to T cells due to its poor antigen-presenting character^31^. Our findings suggest that *Plasmodium* nucleic acid is preferentially taken up by pDCs at first 24 h post YM infection and induce large amount of IFN-α/β production by activating MyD88-IRF7 dependent type I IFN signaling through blocking IL-1β-mediated MyD88-TRAF3-IRF3 signaling and SOCS1 expression in inflammasome-deficient mice. The elevated type I IFN effectively activates cDCs/macrophages and promotes cDCs maturation for efficiently presenting antigens to activate subsequent adaptive immune responses.

During malaria infection, gDNA, RNA, hemozoin, and GPI released from ruptured iRBCs are captured by immune cells^4^. gDNA-hemozoin complex and iRBCs induce TLR9 translocation and NLRP3/AIM2 inflammasomes^14–16,32^. Consistent with the previous findings, we show that the activation of AIM2 and NLRP3 dependent inflammasome in murine primary pDCs, cDCs and macrophages by gDNA and hemozoin during YM infection. Additionally, we also show activation of NLRP3 inflammasome by parasite RNA in pDCs and macrophages, providing new insights of innate immune responses after YM infection. However, other studies show that NLRP3, ASC, Caspase-1, IL-18, or IL-1 receptor have minor or no impact on parasitemia control or mouse survival^15,33,34^. Thus, the role of inflammasome in host resistance to *Plasmodium* infection may be strain-specific^18,19^.

Type I IFN has been reported to regulate inflammasome activation and IL-1β production in response to fungal^25^, bacteria^24,26^ and virus infection^27^. Recent study also shows that inflammasome signaling could dampen type I IFN signaling by cleaving cGAS and decreasing cGAS-STING-dependent type I IFN production during DNA virus infection^35^. In this study, we found that inflammasome-coupled IL-1β-mediated MyD88-TRAF3-IRF3 signaling contributes to induce SOCS1 expression and inhibits MyD88-IRF7 dependent type I IFN in pDCs. To our knowledge, this is the first demonstration to show that inflammasome negatively regulates MyD88-IRF7-dependent type I IFN response to malaria infection.

Malaria infection triggers multiple innate immune response pathways, and the parasites may develop mechanisms to evade host immune responses, including induction of negative regulators to downregulate cytokine production and inhibit activation of immune cells. In this study, we demonstrated that expression of SOCS1 is regulated by both MAVS/STING-dependent type I IFN and inflammasome-coupled IL-1β-mediated MyD88-TRAF3-IRF3 signaling pathways. The MAVS-IRF3-dependent type I IFN provides a dominant effect on SOCS1 expression, and mice lacking MAVS have the highest type I IFN levels and the strongest resistance to YM infection. Meanwhile, inflammasome induced IL-1β production could also upregulate SOCS1 expression and provide a secondary inhibitory mechanism on MyD88-IRF7 dependent type I IFN production. Our results demonstrate the crosstalk between inflammasome and several type I IFN signaling pathways. MAVS/STING-dependent and inflammasome-IL-1β-MyD88-TRAF3-mediated signaling pathways synergize to induce SOCS1 expression, which, in turn, suppress MyD88-IRF7 dependent type I IFN response (Supplementary Fig. 7b), leading to fast parasite growth and host death. MAVS-mediated signaling is more powerful in inducing SOCS1 expression and in inhibiting MyD88-IRF7 dependent type I IFN production, particularly at early hours of infection or stimulation (peak at 6 h), and the effects of inflammasome induced IL-1β signaling on SOCS1 expression may increase with time of infection.

SOCS1 was initially identified as a negative regulator of JAK/STAT-mediated signal cascade initiated by cytokines such as IL-4, IL-6, and IFN-γ^36,37^. SOCS1 also inhibits MyD88-dependent NF-κB signaling by interacting with IRAK and NF-κB, promoting their degradation, thus preventing NF-κB activation^30^. In this and our previous studies^19^, we show that negative regulation of SOCS1 in controlling MyD88-IRF7 dependent type I IFN signaling by interacting with MyD88 is critical to the host resistance to lethal malaria infection. Since SOCS1 could both inhibit MyD88-dependent production of type I IFN and the downstream signaling of IFN-stimulating pathway for IFN-γ production (a JAK1/STAT dependent signaling), we further demonstrate that SOCS1 silencing could not rescue host survival in *Myd88*^−/−^ mice, indicating that SOCS1 controls the host protection mainly through MyD88, but not the downstream signaling of IFN-stimulating pathway. Importantly, SOCS1 could be induced by both IRF3-dependent type I IFN signaling and the downstream signaling of IFN-stimulating pathway, which may sustain its dual function on inhibition of type I IFN production and downstream IFN-γ production.

In summary, our results show that inflammasome-deficient mice are resistant to lethal YM infection by dampening IL-1β signaling and subsequent SOCS1 expression through MyD88-TRAF3-IRF3-dependent type I IFN signaling. Attenuation of SOCS1 relieves its inhibition on MyD88-IRF7 dependent type I IFN production in pDCs. The higher amount of MyD88-IRF7 dependent type I IFN cytokines in pDCs of *Il1r1*^−/−^, *Aim2*^−/−^, *Nlrp3*^−/−^, and *Casp1*^−/−^ mice at the early stage of YM infection facilitates anti-malaria adaptive immune response. Inflammasome-coupled IL-1β-mediated MyD88-TRAF3-IRF3-dependent and MAVS/STING-mediated type I IFN signaling pathways dynamically regulate SOCS1 expression and modulate MyD88-IRF7 dependent type I IFN signaling. Thus, our findings not only discover a critical regulatory mechanism of inflammasome on two type I IFN signaling pathways in pDCs, but also provide potential therapeutic targets for the development of safe and effective malaria vaccines.

## Methods

### Microbes

*Plasmodium yoelii* YM has been previously described^38^.

### Primary cells

Bone marrow cells were isolated from the tibia and femur and cultured in RPMI1640 medium with 10% FBS, 1% penicillin-streptomycin, 55 μM β-mercaptoethanol and 10% L929 conditioned media containing macrophage-colony stimulating factor (M-CSF) for 6 days, 20 ng/ml murine GM-CSF and 10 ng/ml IL-4 for 6–8 days or 200 ng/ml Flt3L for 7 days to harvest BMDMs, cDCs, or pDCs, respectively. pDCs, cDCs and macrophages were purified directly from splenocytes by anti-mPDCA-1, anti-mCD11c and anti-mCD11b microbeads, respectively, following the manufacturer’s instructions.

### Animals

Female mice of C57BL/6 (WT), *Aim2*^−/−^, *Casp1*^−/−^, *Myd88*^−/−^, *Nlrp3*^−/−^*, Il1r1*^−/−^ and *Zbtb46*-DTR mice were purchased from The Jackson Laboratory. *Traf3*^*flox/flox*^ mice were kindly gifted from Dr. Shao-Cong Sun (University of Texas, MD Anderson Cancer Center) and cross with CD11c-cre (The Jackson Laboratory) to generate *Traf3*^*f/f*^ CD11c-cre mice, *Irf3*^−/−^*:Irf7*^−/−^ mice were from Dr. Kate Fitzgerald (University of Massachusetts Medical School) and Dr. Tadatsugo Taniguchi (The University of Tokyo), and crossed with C57BL/6 mice to get *Irf3*^−/−^ mice. For *plasmodium* infection, 0.5 × 10^6^ iRBCs (otherwise, indicated specifically in the figure legend) suspended in 200 µl PBS from the donor mice were intraperitoneally injected into experimental mice. All mouse-related procedures were performed according to experimental protocols approved by the Animal Care and Welfare Committee at Houston Methodist Research Institute and in accordance with NIH-approved animal study protocol LMVR-11E.

### Cell depletion

To deplete pDCs, pDC-depleting functional-grade mAb (anti-mPDCA-1 IgG, clone JF05-1C2.4.1) and the corresponding isotype control IgG were purchased from Miltenyi Biotec (Auburn, CA), and two intraperitoneal injections of antibody (250 µg/mouse) per mouse were administered 12 h prior and after the indicated times. To deplete macrophage, clodronate liposomes (from Dr. Nico. Van Rooijen) were injected intraperitoneally at 750 µg/injection at the indicated times, control liposomes served as control. For cDCs depletion, bone marrow chimeras were reconstituted for at least 6 to 8 weeks after lethal irradiation and i.v. transferred with 10 × 10^6^ bone marrow cells from *Il1r1*^−/−^ *Zbtb46-DTR* mice, then injected with DT at a dose of 2.5 ng per gram of body weight.

### ELISA

Mouse serum or cell supernatants were collected at the indicated time after infection or stimulation and subjected to analysis with commercial ELISA kits for mouse IFN-α, IFN-β, (PBL Biomedical Laboratories) or IFN-γ, IL-6, IL-1β (eBioscience), following the manufacturer’s instructions.

### Isolation of *Plasmodium* gDNA, RNA, and hemozoin

Parasite-infected mice blood was collected in saline solution and filtered to deplete white blood cells. Parasites were spun down after RBC lysis buffer treatment, and the lysate was incubated with buffer A (150 mM NaCl, 25 mM EDTA, 10% SDS and protein kinase) overnight. gDNAs were isolated using phenol/chloroform extraction, and RNAs were isolated using TRIzol reagent (Invitrogen). Hemozoin were purified as previously described^32^.

### RNA preparation and qPCR

Total RNA was harvested from splenic tissue, lymph node, or stimulated cells using the TRIzol reagent (Invitrogen), and the complimentary cDNA was generated using reverse transcriptase IV (Invitrogen). Real-time PCR was performed using the ABI Prism 7000 analyzer (Applied Biosystems) and iTaq SYBR Green Supermix (BioRad) with specific primers.

### Immunoblot analyze

For immunoblotting analysis, whole-cell extracts were lysed with low-salt lysis buffer, and supernatants were precipitated with methanol/chloroform, boiled for 5 min with SDS loading buffer (Cell Signaling Technology, Danvers, MA, USA) and resolved on SDS-PAGE gels. The proteins were transferred to PVDF membranes (BioRad) and further incubated with the indicated antibodies. LumiGlo Chemiluminescent Substrate System from KPL (Gaithersburg, MD) was used for protein detection. Images were cropped for presentation and the full size images are presented in Supplementary Fig. 8.

### Quantification and statistical analysis

All analyses were performed using GraphPad Prism version 5.0 (GraphPad Software, La Jolla, CA). Data are presented as means ± s.d., unless otherwise stated. Differences in mice survival were evaluated with Mantel–Cox log-rank test. The sample size for each experiment, *n*, is included in the results section and the associated figure legend. Statistical significance of differences between two groups was assessed by unpaired Student *t* tests and a *p* value of <0.05 was considered significant.

## Electronic supplementary material

Supplementary Information

Reporting Summary

## Data Availability

Data are available from the corresponding authors upon reasonable request.
