# Supplementary material for: Inflammasome activation negatively regulates MyD88-IRF7 type I IFN signaling and anti-malaria immunity
Source: Nat Commun. 2018 Nov 23;9:4964. doi: 10.1038/s41467-018-07384-7 (PMC6251914; doi:10.1038/s41467-018-07384-7)
Supplement: Supplementary file 1 — Supplementary Information [file 41467_2018_7384_MOESM1_ESM.pdf]

Inflammasome activation negatively regulates MyD88-IRF7 type I IFN signaling and anti-malaria immunity

Yu et al.

Supplementary Figure 1

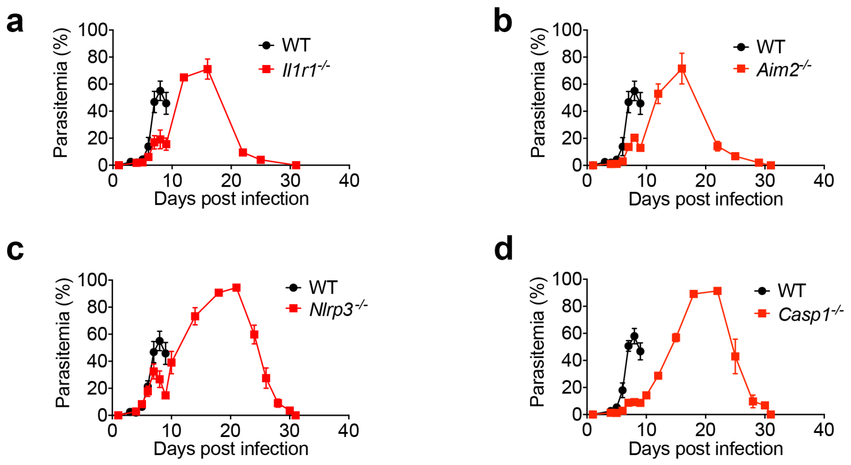

Supplementary Figure 1. Inflammasome gene deficient mice clear *plasmodium* at late stage of *P. yoelii* YM infection. *Il1r1*$^{-/-}$ (a), *Aim2*$^{-/-}$ (b), *Nlrp3*$^{-/-}$ (c), *Casp1*$^{-/-}$ (d) and WT mice (n=5) were intraperitoneally infected with *P. yoelii* YM (0.5×10^6 iRBCs). Daily parasitemias (up to 30 days) are shown. Data are representative of three independent experiments.

Supplementary Figure 2

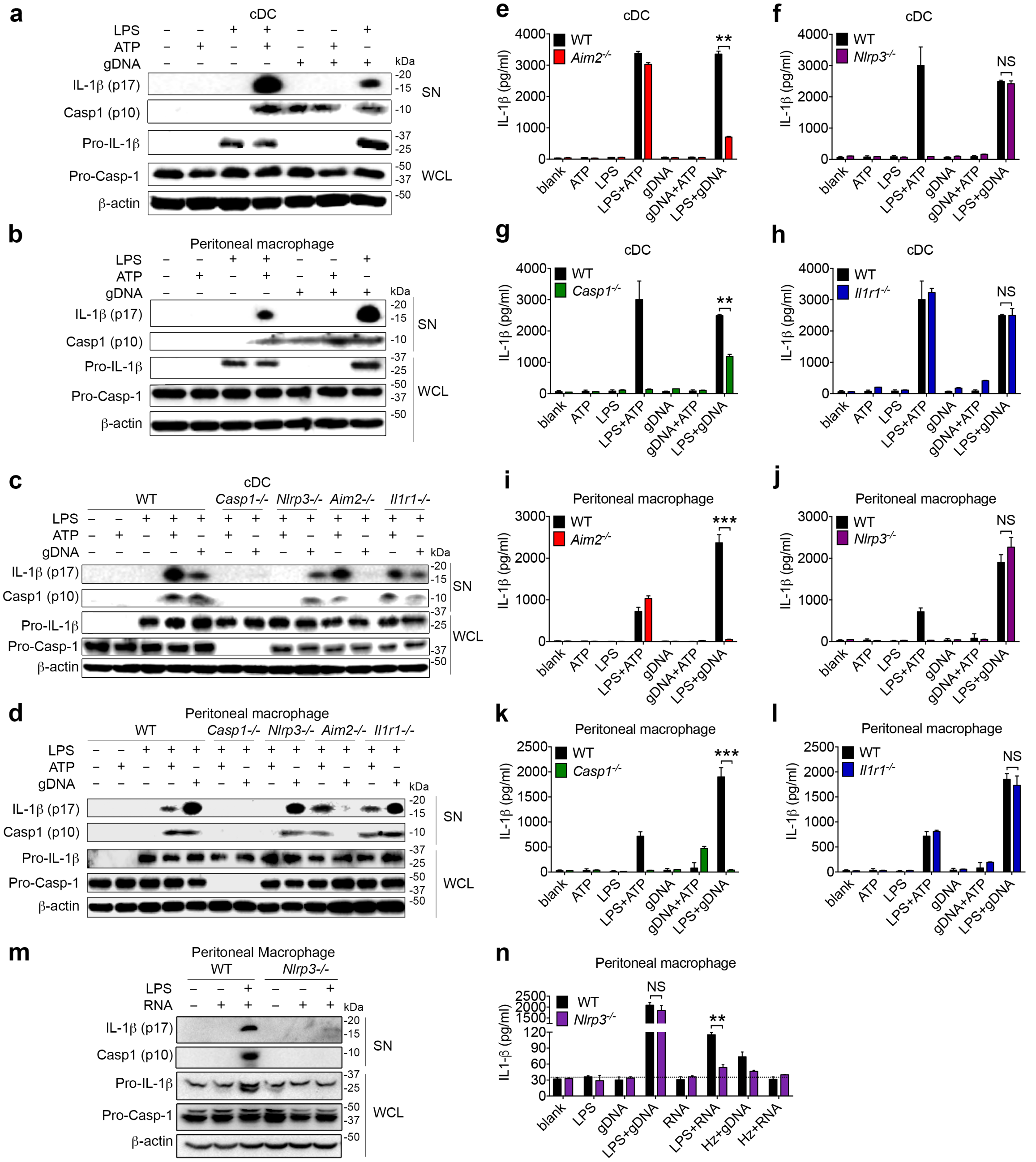

Supplementary Figure 2. *Plasmodium* gDNA, RNA and hemozoin induce IL-1β production through activation of AIM2 and NLRP3 inflammasome in cDCs and peritoneal macrophages. (a-b) WT cDCs (a) and peritoneal macrophages (b) were stimulated as indicated for 24 h, cell lysate and supernatants were collected for immunoblotting analysis. (c-d) WT, *Casp1*$^{-/-}$, *Nlrp3*$^{-/-}$, *Aim2*$^{-/-}$ and *Il1r1*$^{-/-}$ cDCs (c) and peritoneal macrophages (d) were stimulated as indicated (LPS primed for 3 h and gDNA stimulation for 24 h). Cell lysate and supernatants were collected for immunoblotting analysis. (e-h) WT, *Aim2*$^{-/-}$ (e), *Nlrp3*$^{-/-}$ (f), *Casp1*$^{-/-}$ (g) and *Il1r1*$^{-/-}$ (h) cDCs were stimulated as indicated (LPS primed for 3 h and gDNA stimulation for 24 h),

supernatants were collected for ELISA. (i-l) WT, *Aim2*$^{-/-}$ (i), *Nlrp3*$^{-/-}$ (j), *Casp1*$^{-/-}$ (k) and *Il1r1*$^{-/-}$ (l) peritoneal macrophages were stimulated as indicated (LPS primed for 3 h and gDNA stimulation for 24 h), supernatants were collected for ELISA. (m-n) WT and *Nlrp3*$^{-/-}$ peritoneal macrophages were stimulated as indicated. Cell lysate were collected for immunoblotting analysis (m) and supernatants were collected for immunoblotting analysis (m) and ELISA (n). Data are representative of three independent experiments and are plotted as the mean ±s.d. $^{*}p<0.05$, $^{**}p<0.01$, $^{***}p<0.001$ vs. corresponding control. NS, not significant.

Supplementary Figure 3

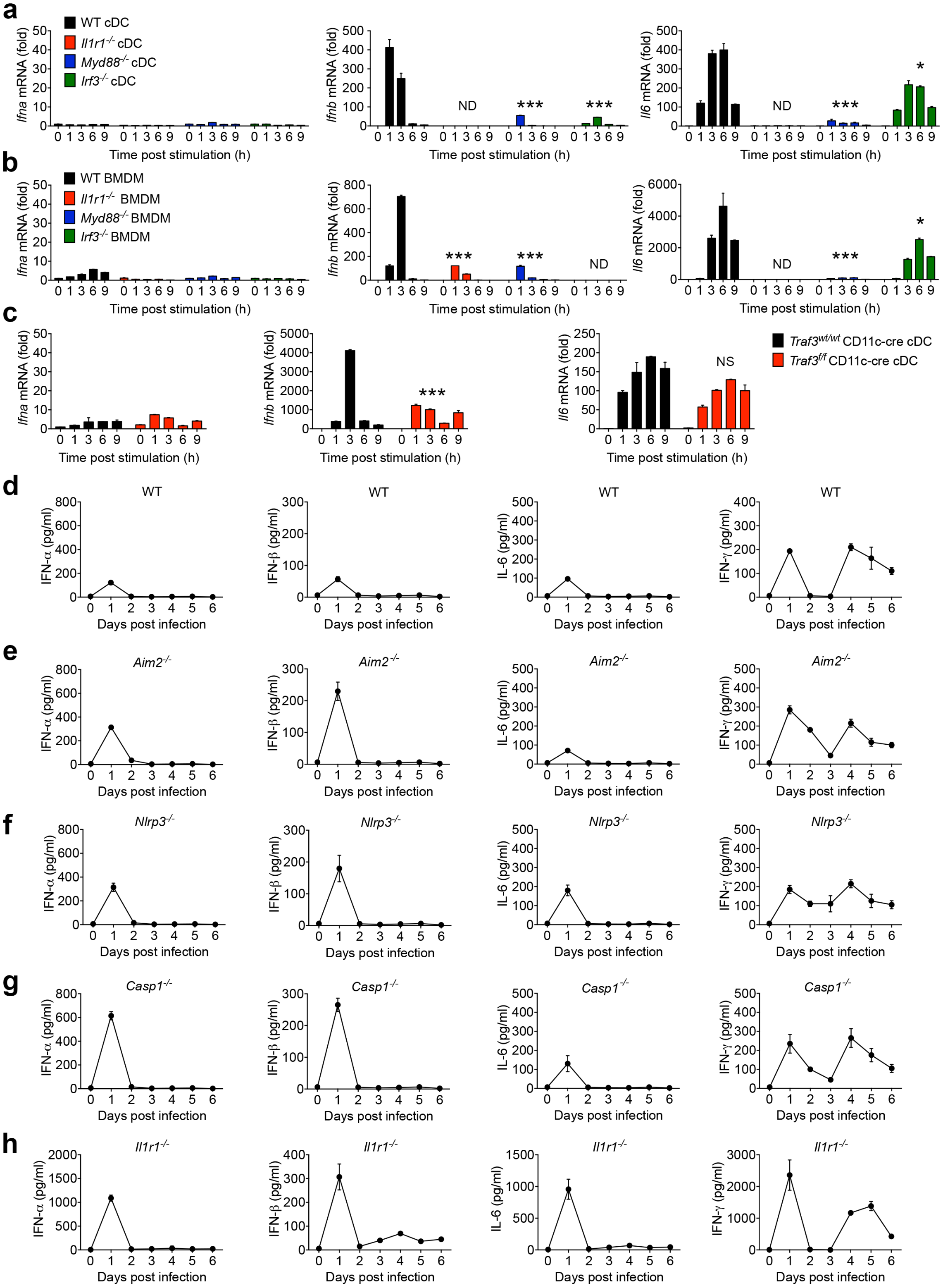

Supplementary Figure 3. Production of type I IFN, IL-6 and IFN-γ in WT and inflammasome-defective cells or mice (n=5) after *in-vitro* stimulation or *in-vivo* infection. (a) WT, *Il1r1*$^{-/-}$, *Myd88*$^{-/-}$ and *Irf3*$^{-/-}$ cDCs were stimulated with recombinant mouse IL-1β for indicated times, and RNA from cDCs was isolated and used for expression analysis of *Ifna, Ifnb* and *Il6* by using qPCR. (b) WT, *Il1r1*$^{-/-}$, *Myd88*$^{-/-}$ and *Irf3*$^{-/-}$ BMDM were stimulated with recombinant mouse IL-1β for indicated times, and RNA from BMDM was isolated and used for expression analysis of *Ifna, Ifnb* and *Il6* by using qPCR. (c) *Traf3*$^{wt/wt}$ *CD11c-cre* and *Traf3*$^{f/f}$ *CD11c-cre* cDCs were stimulated with recombinant mouse IL-1β for indicated times, and RNA from cells was isolated and used for expression analysis of *Ifna, Ifnb* and *Il6* by using qPCR. (d-h) WT (d), *Aim2*$^{-/-}$ (e), *Nlrp3*$^{-/-}$ (f), *Casp1*$^{-/-}$ (g), and *Il1r1*$^{-/-}$ (h) mice (n=5) were intraperitoneally infected with *P. yoelii* YM. Serum was collected at indicated times and subjected to ELISA analysis of IFN-α, IFN-β, IL-6 and IFN-γ. Data are representative of three independent experiments. $^{*}p<0.05$, $^{**}p<0.01$, $^{***}p<0.001$ vs. corresponding control. NS, not significant. ND, not detected.

Supplementary Figure 4

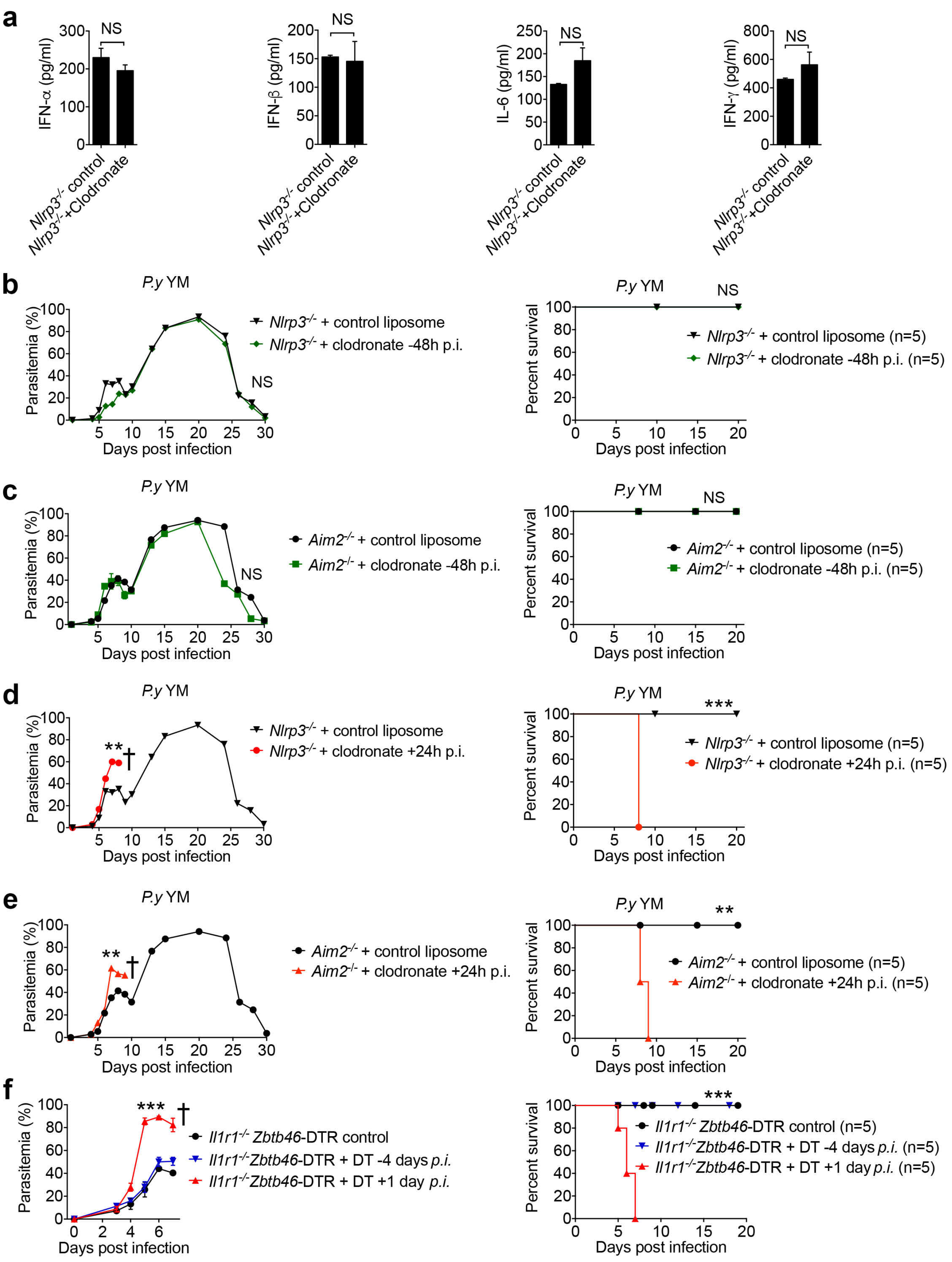

Supplementary Figure 4. Macrophages and cDCs play fundamental roles at late time after *P. yoelii* YM infection in inflammasome related deficient mice. (a) *Nlrp3*$^{-/-}$ mice (n=5) were pretreated with clodronate at 2 days before infection, and then infected with *P. yoelii* YM. Serum was collected at 24 h post infection and subjected to ELISA analysis of IFN-α, IFN-β, IL-6 and IFN-γ. (b-c) *Nlrp3*$^{-/-}$ (b) and *Aim2*$^{-/-}$ (c) mice (n=5) were pretreated with clodronate at 2 days before infection, and then infected with *P. yoelii* YM. Parasitemias and survivals were monitored daily. (d-e) *Nlrp3*$^{-/-}$ (d) and *Aim2*$^{-/-}$ (e) mice (n=5) were infected with *P. yoelii* YM, followed by clodronate injection at day 1 post infection. Parasitemias and survivals were monitored daily. (f) WT chimeric mice (n=5) were irradiated and transplanted with bone marrow cells of *Il1r1*$^{-/-}$ *Zbtb46*-DTR mice, then untreated or treated with DT as indicated at 4 days before or 1 day after *P. yoelii* YM infection. Daily parasitemias and mortality rates are shown. Data are representative of three independent experiments and are plotted as the mean ±s.d. $^{**}p<0.01$, $^{***}p<0.001$ *vs.* corresponding control. NS, not significant. † denotes mouse death.

Supplementary Figure 5

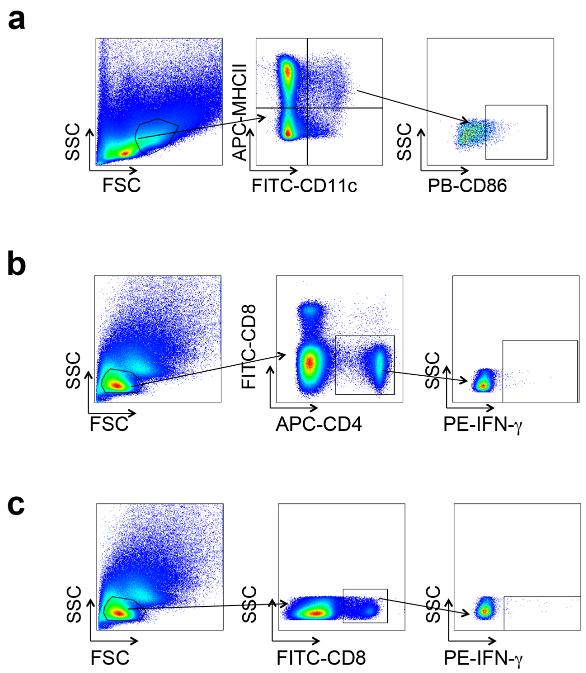

Supplementary Figure 5. Gating strategies used for FACS data. (a) Gating strategy for CD86$^+$ cells in CD11c$^+$MHC-II$^+$ cells from splenocytes of WT and *Il1r1*$^{-/-}$ mice (n=5) for surface staining presented on Fig. 5j. (b) Gating strategy for IFN-γ$^+$ cells in CD4$^+$ cells from splenocytes of WT and *Il1r1*$^{-/-}$ mice (n=5) for intracellular staining presented on Fig. 5l. (c) Gating strategy for IFN-γ$^+$ cells in CD8$^+$ cells from splenocytes of WT and *Il1r1*$^{-/-}$ mice (n=5) for intracellular staining presented on Fig. 5m.

Supplementary Figure 6

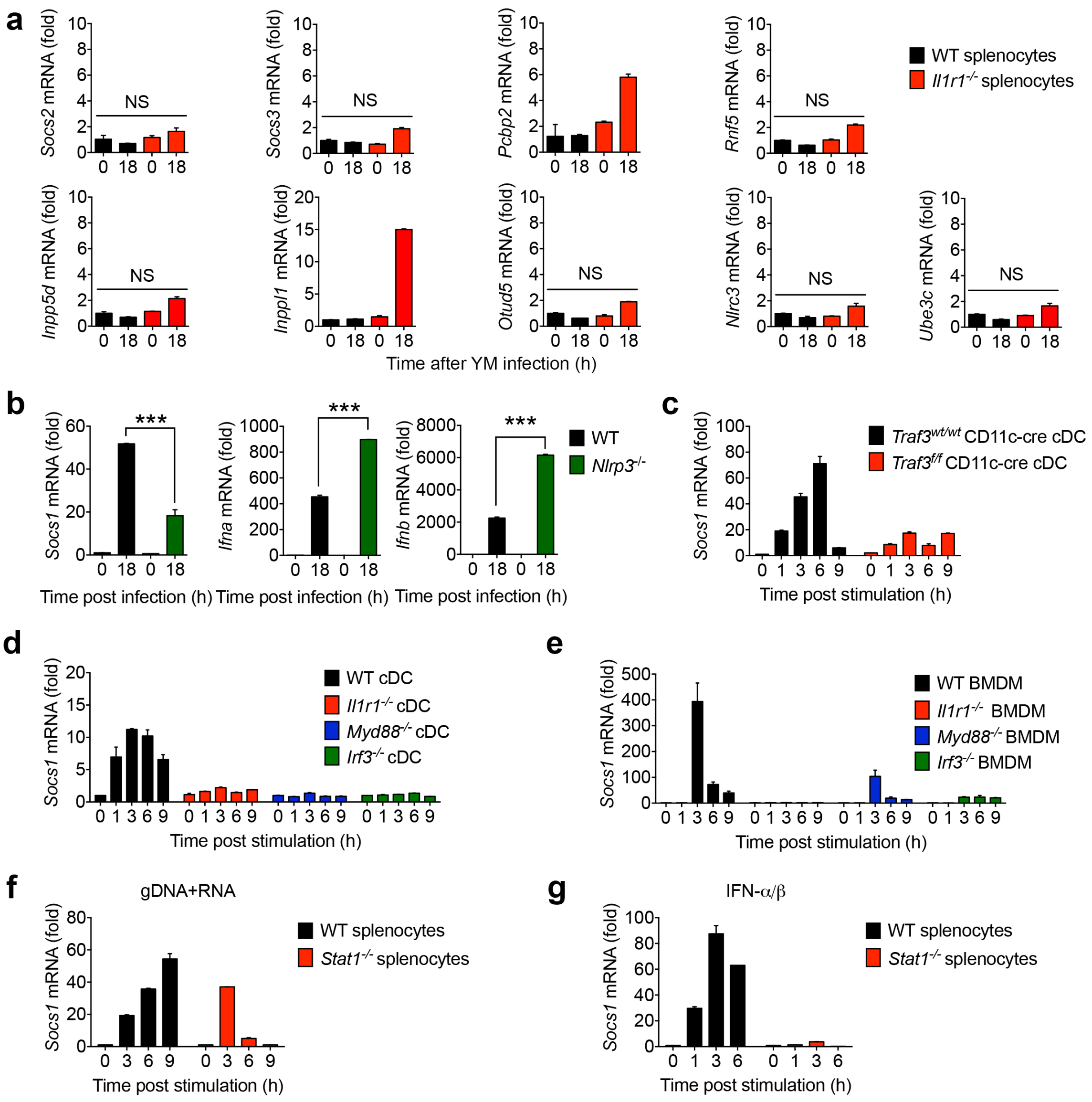

Supplementary Figure 6. Negative regulator SOCS1 is induced in a MyD88-TRAF3-IRF3 dependent manner. (a) Expression of putative negative regulators *Socs2, Socs3*, *Pcbp2*, *Rnf5, Inpp5d*, *Inppl1*, *Otud5*, *Nlrc3* and *Ube3c* in the splenocytes of WT and *Il1r1*$^{-/-}$ mice (n=5) at the indicated times after *P. yoelii* YM infection. RNA from splenocytes was isolated and used for expression analysis by using qPCR. (b) WT and *Nlrp3*$^{-/-}$ mice (n=5) were infected with *P. yoelii* YM for indicated times, and RNA from splenocytes was isolated and used for expression analysis of *Socs1*, *Ifna* and *Ifnb* by using qPCR. (c) WT and *Traf3*$^{f/f}$ *CD11c-cre* cDCs were stimulated with recombinant IL-1β (2 μg/ml) for indicated times, RNA from cDCs was isolated and used for expression analysis of *Socs1* by using qPCR. (d-e) WT, *Il1r1*$^{-/-}$, *Myd88*$^{-/-}$ and *Irf3*$^{-/-}$ cDCs (d) and BMDM (e) were stimulated with recombinant IL-1β (2 μg/ml) for indicated times, RNA from cDCs or BMDM was isolated and used for expression analysis of *Socs1* by using qPCR. (f-g) WT and *Stat1*$^{-/-}$ splenocytes were stimulated with YM gDNA plus RNA (f) or

recombinant mouse IFN-α/β (g) for indicated times. RNA from splenocytes was isolated and used for expression analysis of *Socs1* by using qPCR. Data are representative of three independent experiments and are plotted as the mean ±s.d. $^{***}p<0.001$ vs. corresponding control. NS, not significant.

Supplementary Figure 7

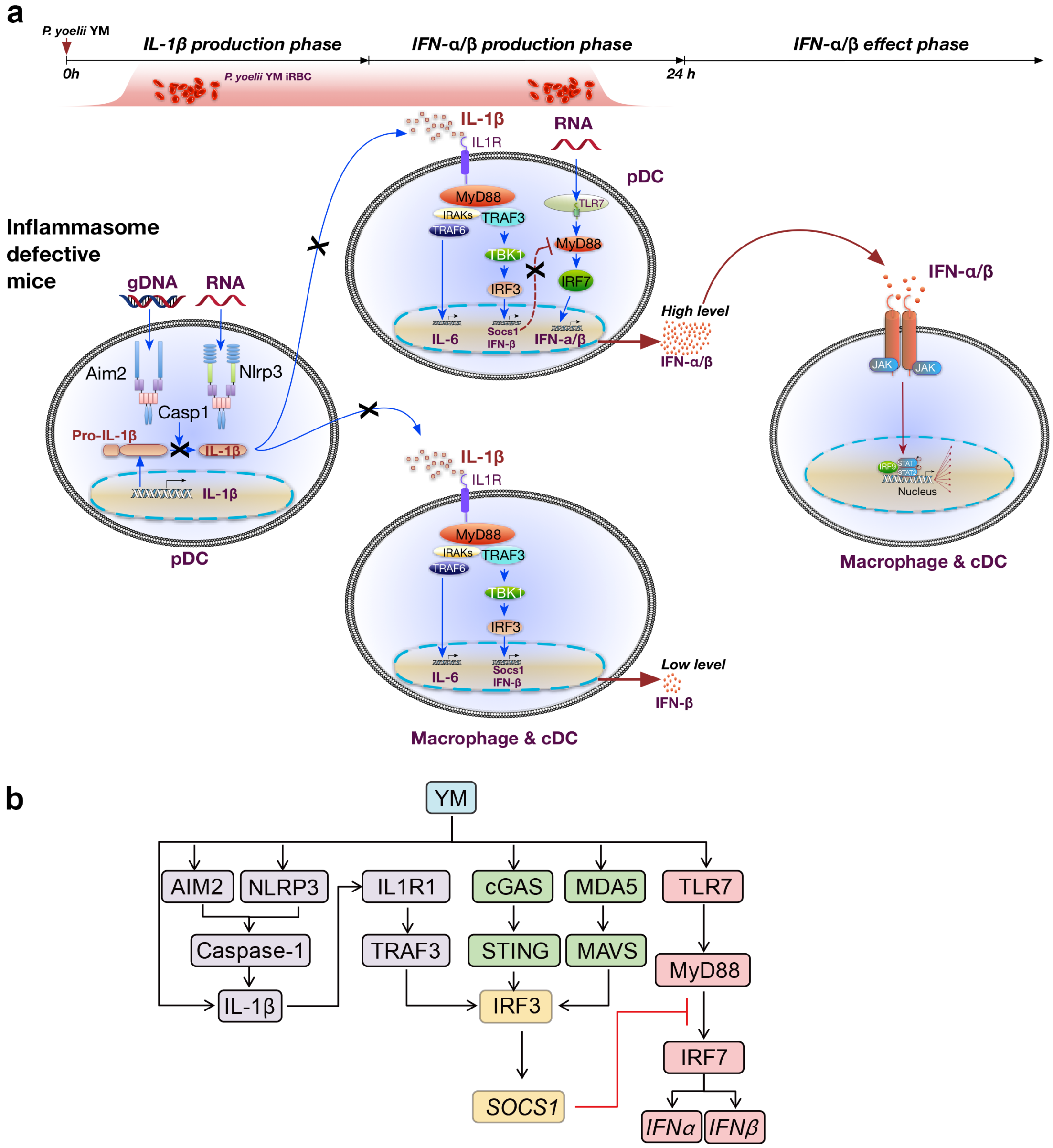

Supplementary Figure 7. A working model to illustrate the cross-regulation between inflammasome coupled IL-1β mediated MyD88-TRAF3-IRF3 type I IFN signaling and MyD88-IRF7 dependent type I IFN signaling after lethal malaria challenging. (a) A working model to illustrate how inflammasome sensors (AIM2 and NLRP3) detect malaria infection and activate IL-1 signaling which regulates MyD88-IRF7 dependent type I IFN signaling pathway post YM infection. Importantly, AIM2 and NLRP3-induced CASP1-dependent inflammasome signaling induces the release of IL-1β in pDCs and activates IL-1 signaling, which induces negative regulator SOCS1 in a MyD88-TRAF3-IRF3 dependent manner and inhibits MyD88-IRF7 dependent type I IFN signaling pathway in WT pDCs in response to lethal *P. yoelii* YM infection. Deficiency in AIM2, NLRP3, CASP1 or IL1R1 markedly impairs IL-1 signaling and decreases SOCS1

expression, thus relieves its inhibition on MyD88-IRF7 dependent type I IFN signaling and increases IFN-α and IFN-β production at the early stage of infection (24 h) in response to lethal *P. yoelii* YM infection. (b) Schematic representation of MAVS-, STING-, and inflammasome-IL-1R1-MyD88-TRAF3 mediated SOCS1 expression to inhibit MyD88-IRF7 dependent type I IFN signaling in pDCs.

Supplementary Figure 8
Raw image_Fig 1 (1)

1b-splenocytes_Pro-IL-1β

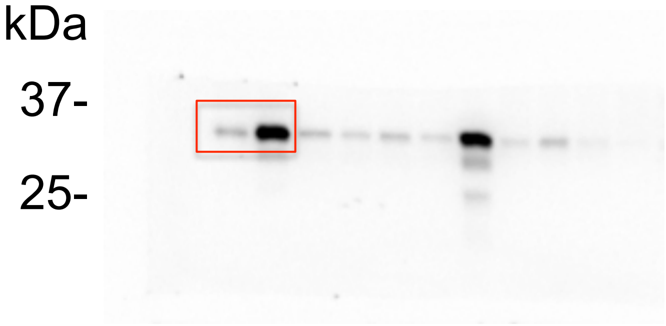

1b-splenocytes_IL-1β (p17)

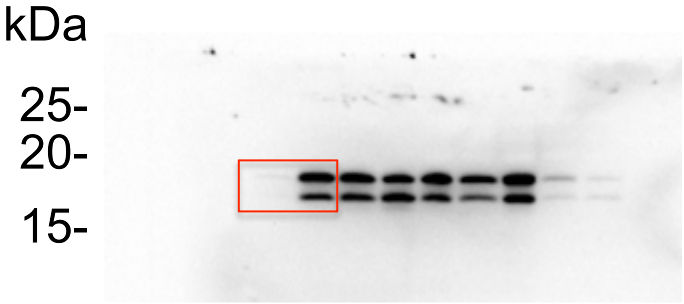

1b-splenocytes_Pro-Casp-1 and Casp-1 (p10)

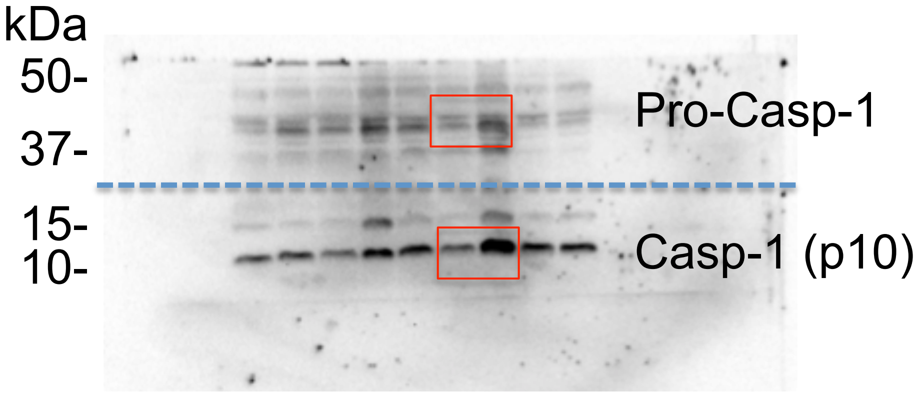

1b-splenocytes_β−actin

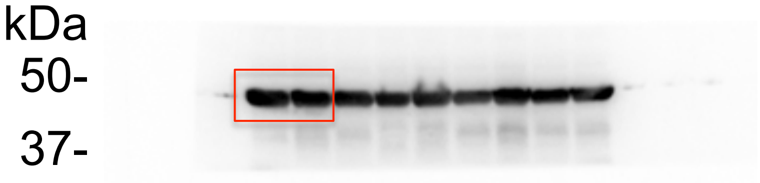

Raw image_Fig 1 (2)

1b-lymph nodes_Pro-IL-1β

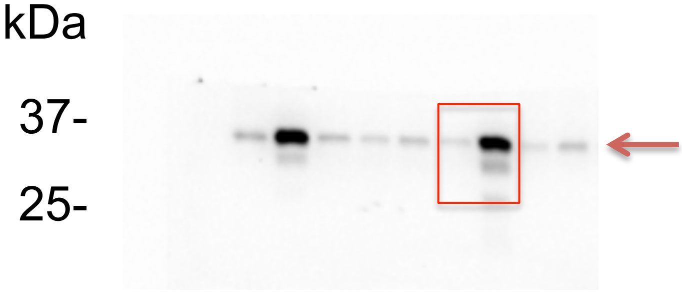

1b-lymph nodes_IL-1β (p17)

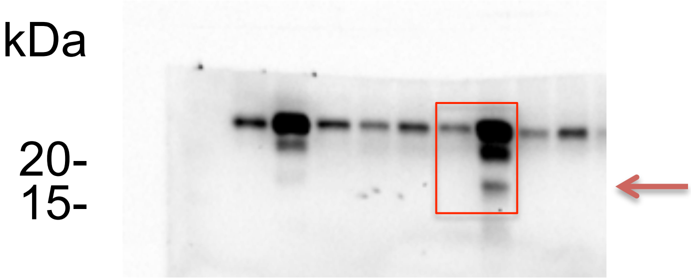

1b-lymph nodes_Pro-Casp-1

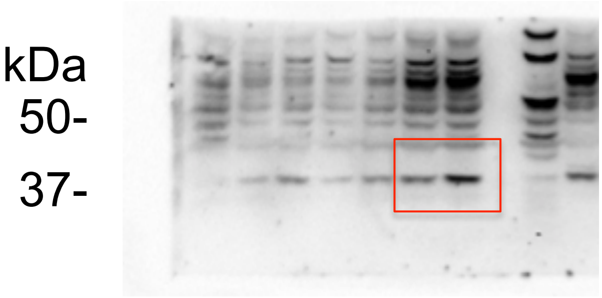

1b-lymph nodes_Casp-1 (p10)

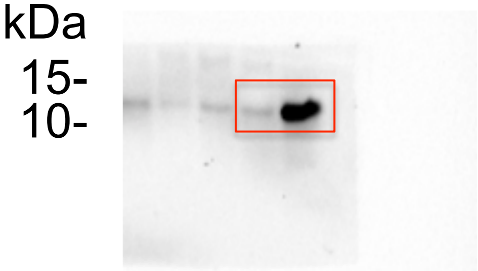

1b-lymph nodes_β−actin

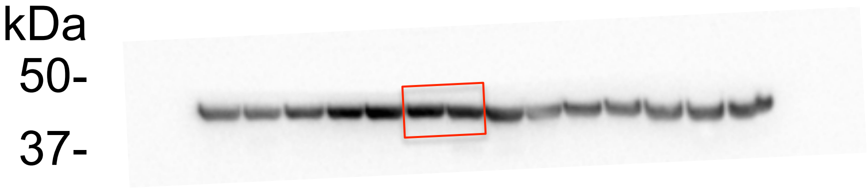

Raw image_Fig 2 (1)

2a_IL-1β (p17)

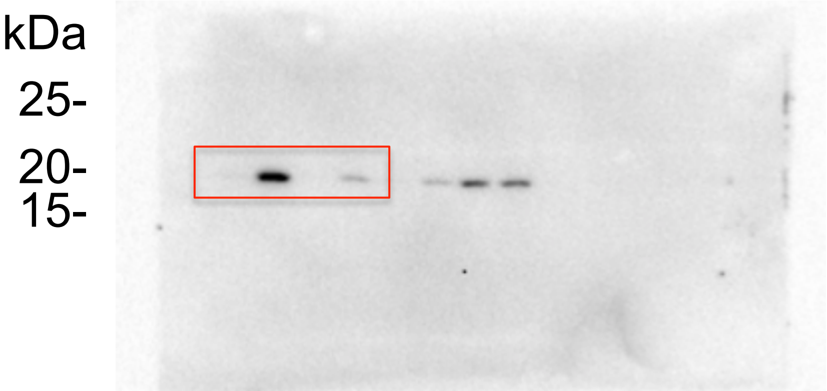

2a_Casp-1 (p20)

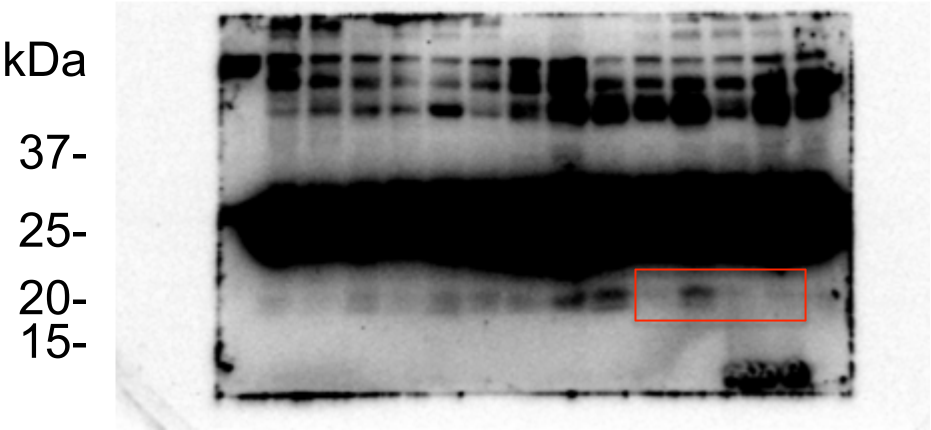

2a_Pro-IL-1β

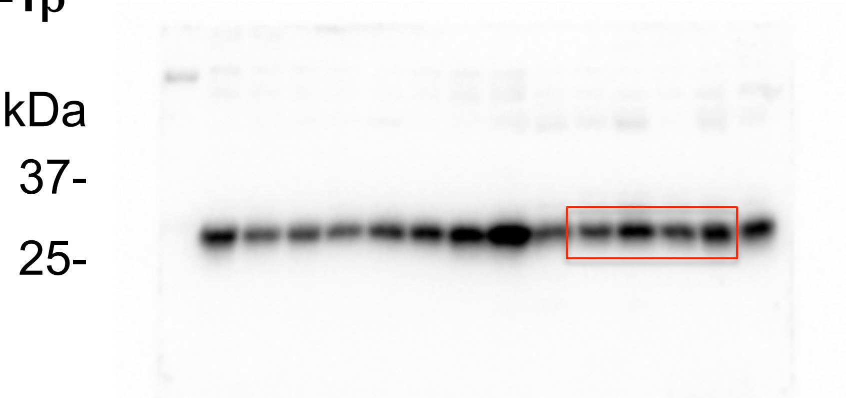

2a_β−actin

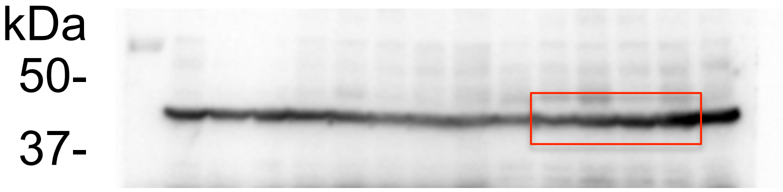

Raw image_Fig 2 (2)

2b_IL-1β (p17)

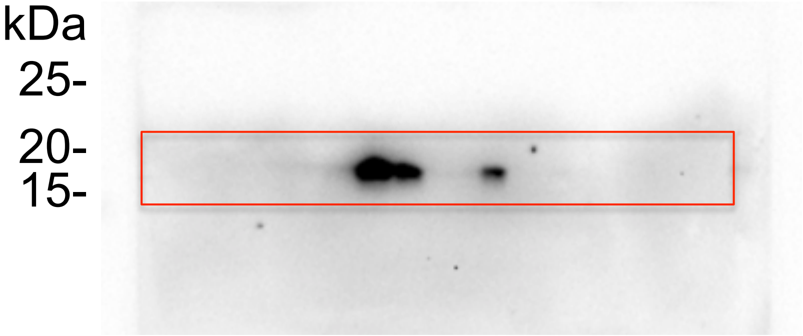

2b_Casp-1 (p20)

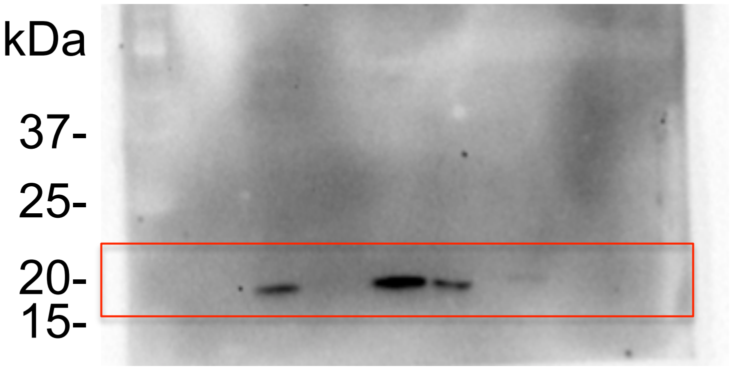

2b_Pro-IL-1β

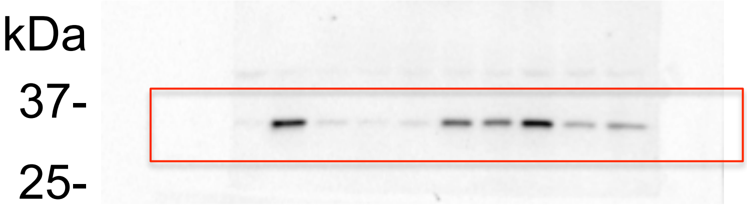

2b_Pro-Casp-1

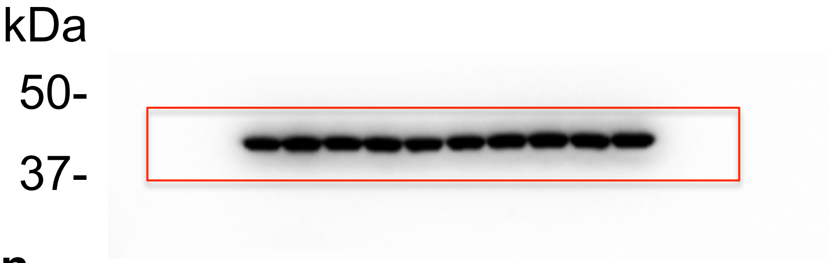

2b_β−actin

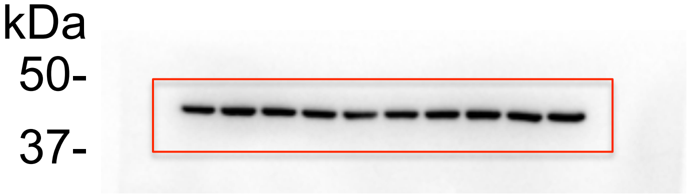

Raw image_Fig S2 (1)

S2a_IL-1β (p17)

kDa
20-
15-

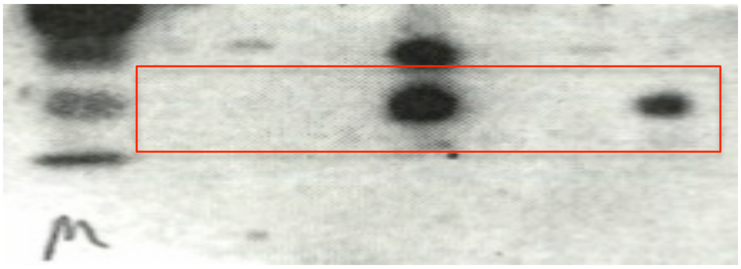

S2a_Casp-1 (p10)

kDa
20-
15-
10-

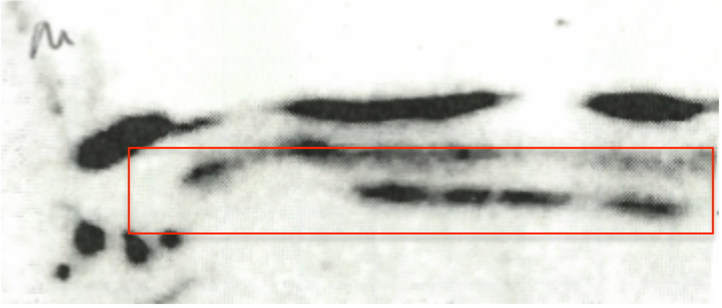

S2a_Pro-IL-1β

kDa
37-
25-

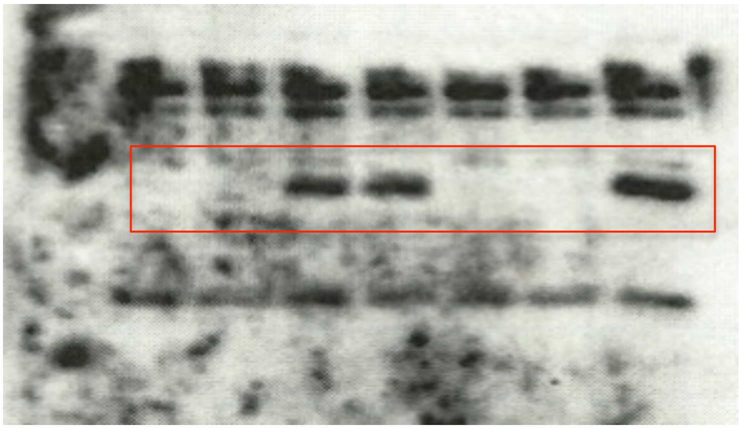

S2a_Pro-Casp-1

kDa
50-
37-

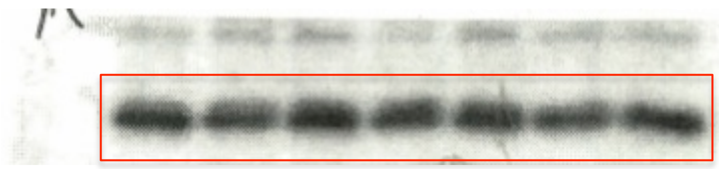

S2a_β−actin

kDa
50-
37-

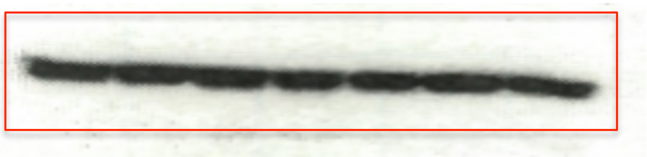

Raw image_Fig S2 (2)

S2b_IL-1β (p17)

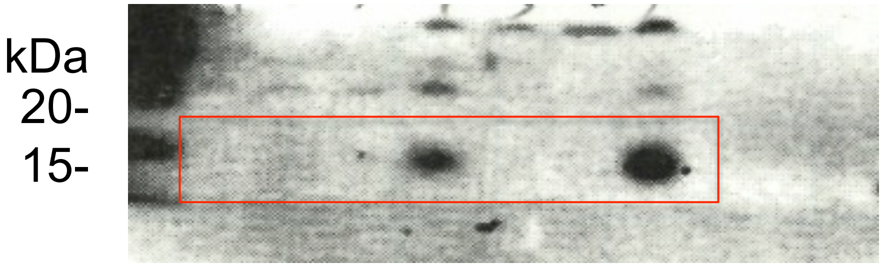

S2b_Casp-1 (p10)

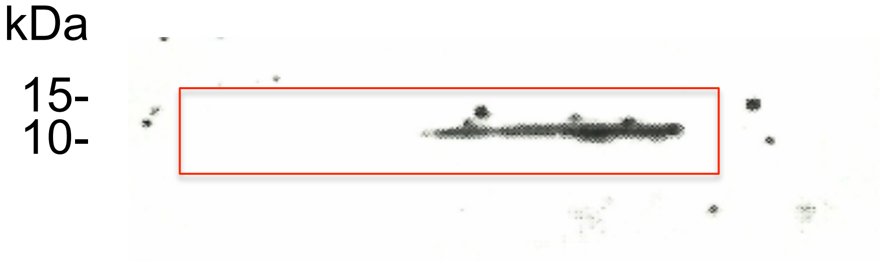

S2b_Pro-IL-1β

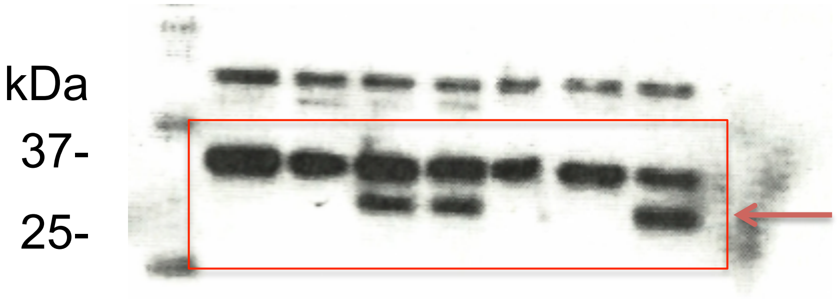

S2b_Pro-Casp-1

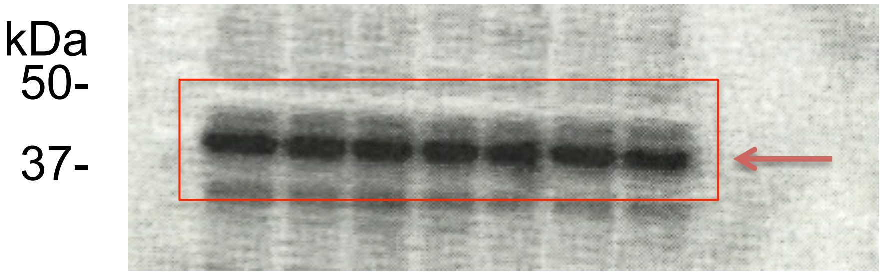

S2b_β−actin

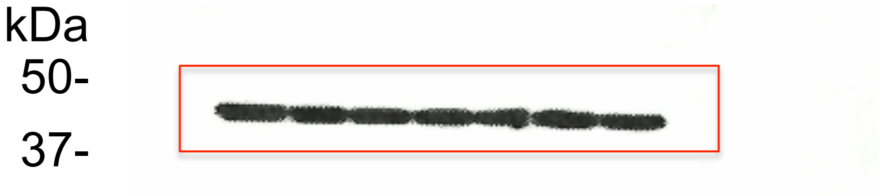

Raw image_Fig S2 (3)

S2c_IL-1β (p17)

kDa
20-
15-

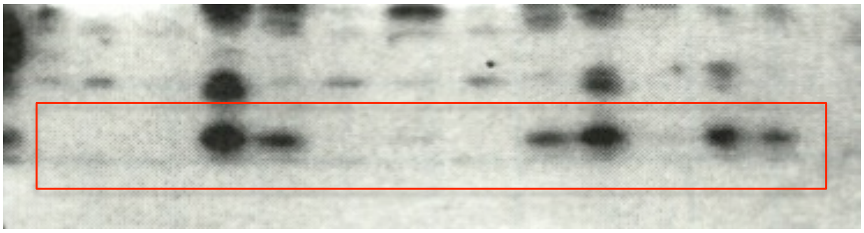

S2c_Casp-1 (p10)

kDa
15-
10-

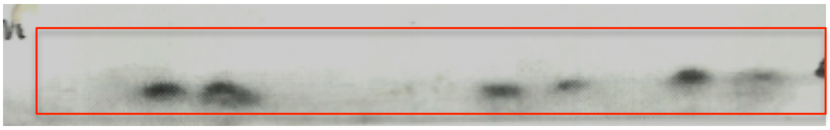

S2c_Pro-IL-1β

kDa
37-
25-

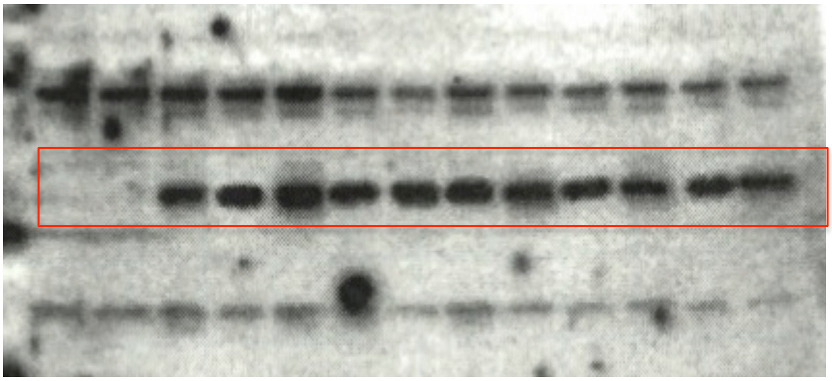

S2c_Pro-Casp-1

kDa
50-
37-

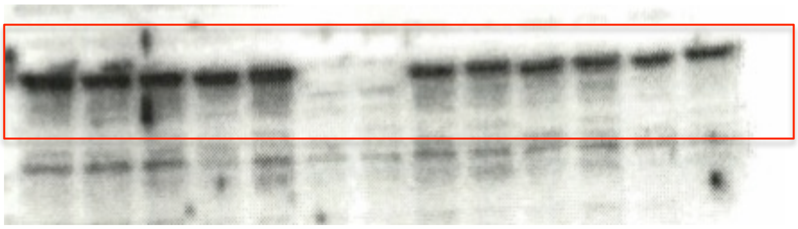

S2c_β−actin

kDa
50-
37-

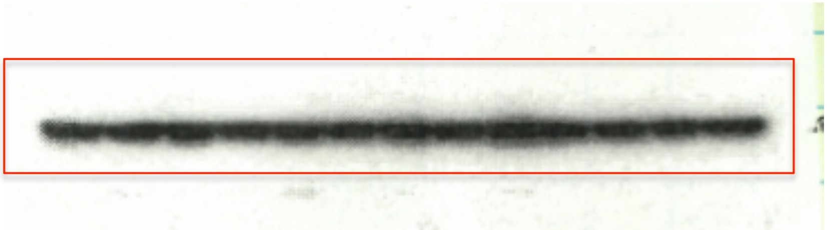

Raw image_Fig S2 (4)

S2d_IL-1β (p17)

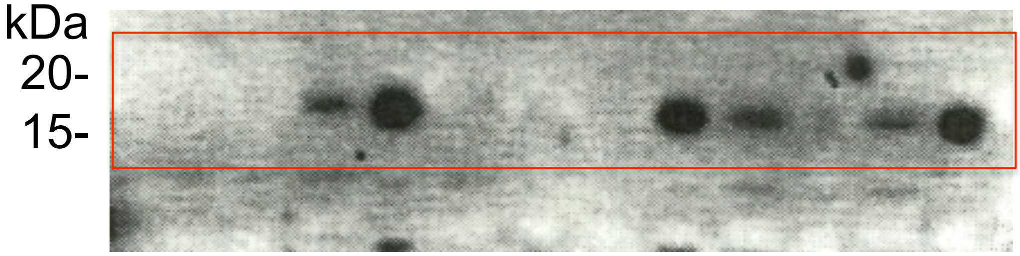

S2d_Casp-1 (p10)

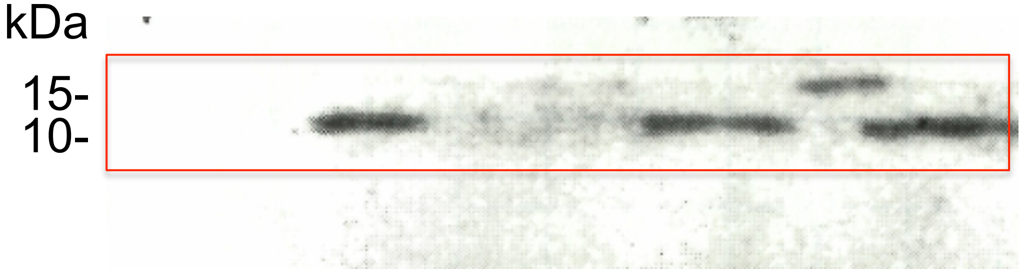

S2d_Pro-IL-1β

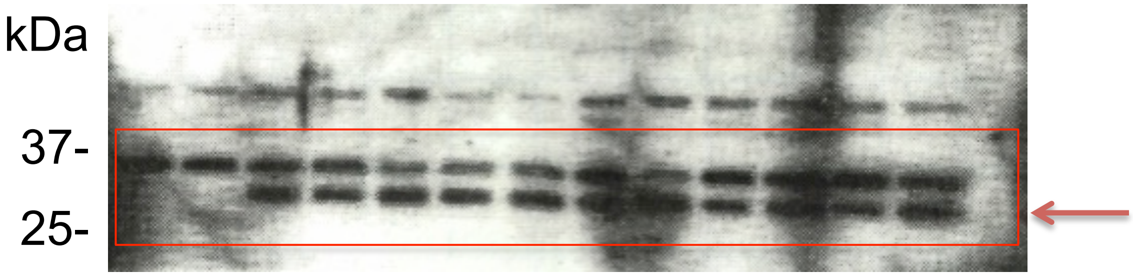

S2d_Pro-Casp-1

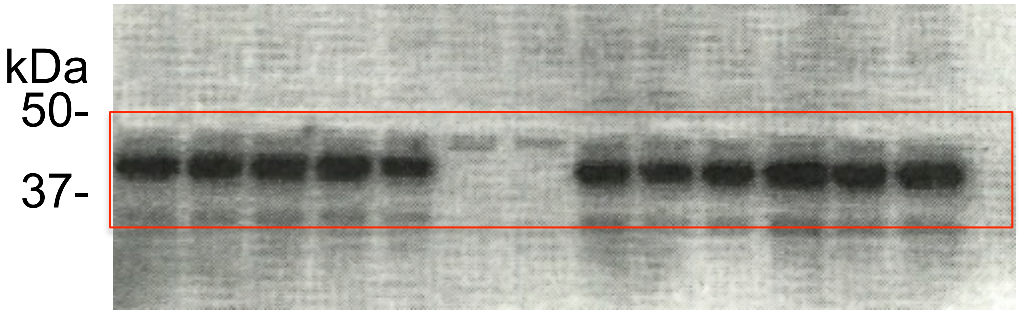

S2d_β−actin

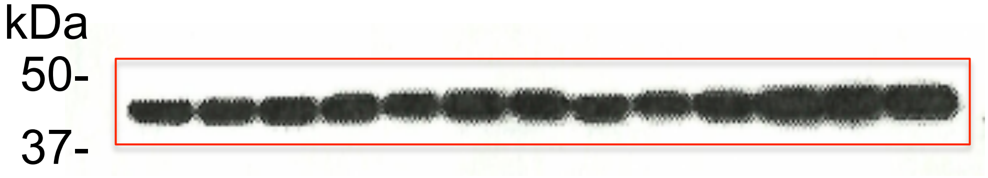

Raw image_Fig S2 (5)

S2m_IL-1β (p17)

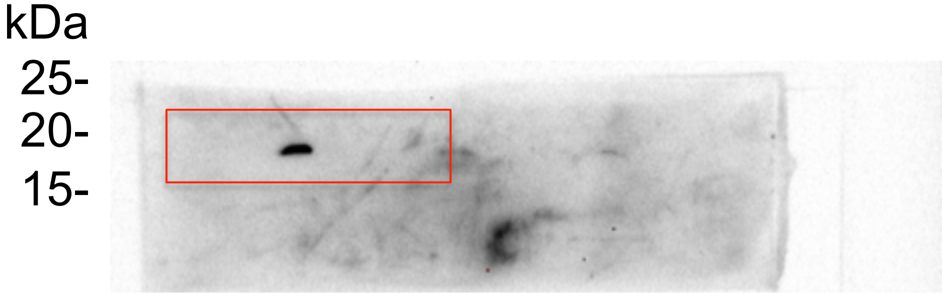

S2m_Casp-1 (p10)

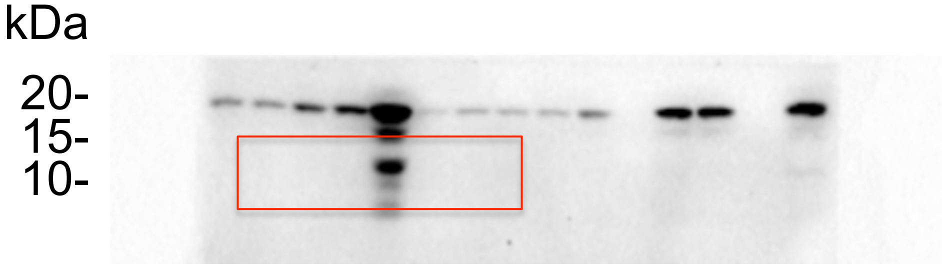

S2m_Pro-IL-1β

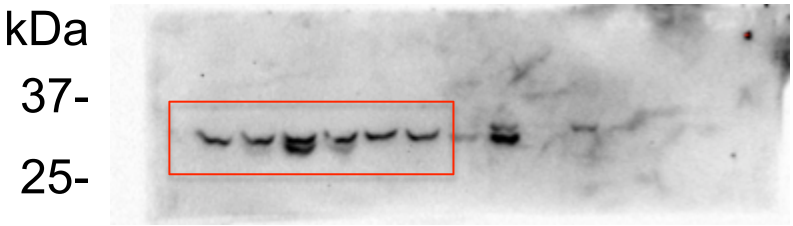

S2m_Pro-Casp-1

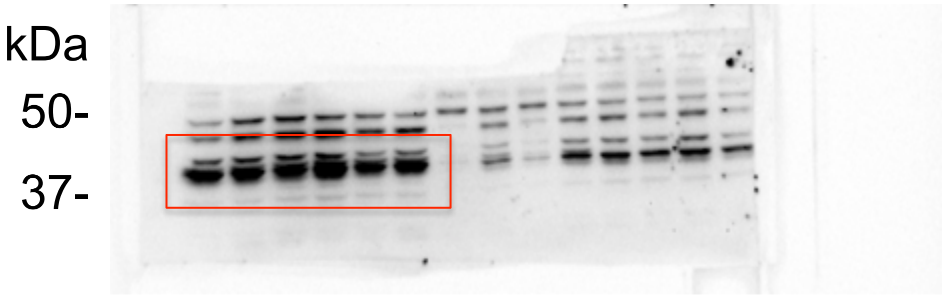

S2m_β−actin

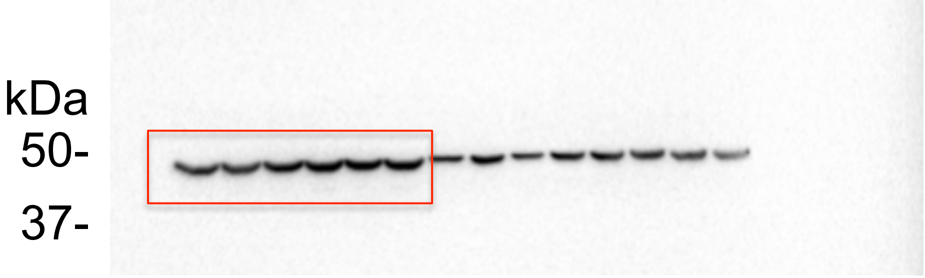

Raw image_Fig 5

5a_18S rRNA

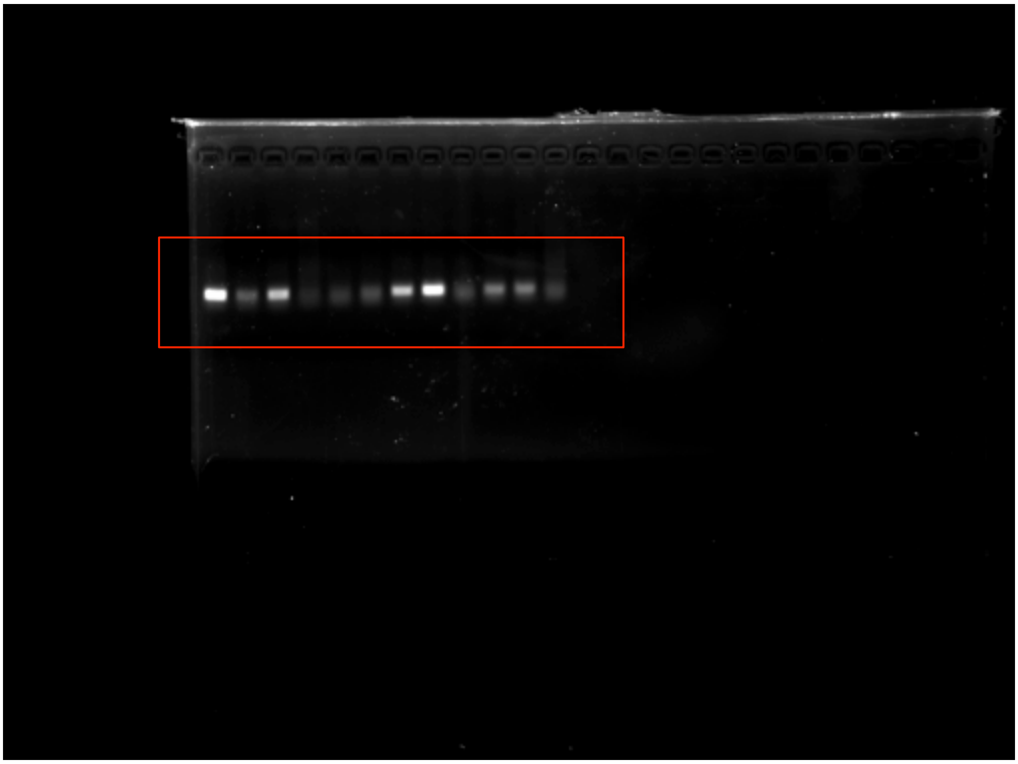

Supplementary Table 1. Primers Used for Real-time PCR

Description gene/protein	Upper primer (5'-3')	Lower primer (5'-3')	Species
IFN-α	GGACTTTGGATTCCCGCAGGAGAAG	GCTGCATCAGACAGCCTTGCAGGTC	Mouse
IFN-β	TCACCTACAGGGCGGACTTC	TCTCTGCTCGGACCACCATC	Mouse
Socs1	CTGCGGCTTCTATTGGGGAC	AAAAGGCAGTCGAAGGTCTCG	Mouse
Socs2	AGTTCGCATTCAGACTACCTACT	TGGTACTCAATCCGCAGGTTAG	Mouse
Socs3	ATGGTCACCCACAGCAAGTTT	TCCAGTAGAATCCGCTCTCCT	Mouse
Inpp5d/Ship1	GCCCCTGCATGGGAAATCAA	TGGGTAGCTGGTCATAACTCC	Mouse
Inppl1/Ship2	CAGCCTGGTATCACCGTGAC	GCCACGCTCTCGCTATCTC	Mouse
Otud5/Duba	CAGTGAAGACGAGTATGAAGCTG	AGCCCGAAATAGACAGGCAC	Mouse
Gsk3b/Gsk3β	TGGCAGCAAGGTAACCACAG	CGGTTCTTAAATCGCTTGTCCTG	Mouse
Nlrc3	CAGATTGGTAACAAAGGAGCCA	CGTTCGGTTTATCTTCAGAGCA	Mouse
Pcbp2	GCCAGATTTGACCAAGCTGC	GAGCTGGATTCAATGCCACTG	Mouse
Raul	TCGCTGATCTCCATACATGACA	CATCGTGACAGCAGGATTAACT	Mouse
Rnf5	CAAGAATGCCCGGTGTGTAAA	GGGTGGAGTTTTCAATCTGGGA	Mouse
GAPDH	AAGGTCATCCCAGAGCTGAA	CTGCTTCACCACCTTCTTGA	Mouse
IL-1b	CACAGCAGCACATCAACAAG	GTGCTCATGTCCTCATCCTG	Mouse
IL-6	CTCTGGGAAATCGTGGAAAT	CCAGTTTGGTAGCATCCATC	Mouse
IL-18	GACTCTTGCGTCAACTTCAAGG	CAGGCTGTCTTTTGTCAACGA	Mouse
18S rRNA	CTTGGCTCCGCCTCGATAT	TCAAAGTAACGAGAGCCCAATG	Rodent
