# Supplementary material for: Inflammasome activation negatively regulates MyD88-IRF7 type I IFN signaling and anti-malaria immunity
Source: Nat Commun. 2018 Nov 23;9:4964. doi: 10.1038/s41467-018-07384-7 (PMC6251914; doi:10.1038/s41467-018-07384-7)
Supplement: Supplementary file 2 — Reporting Summary [file 41467_2018_7384_MOESM2_ESM.pdf]

nature research

Corresponding author(s): Rongfu Wang

Reporting Summary

Nature Research wishes to improve the reproducibility of the work that we publish. This form provides structure for consistency and transparency in reporting. For further information on Nature Research policies, see Authors & Referees and the Editorial Policy Checklist.

Statistical parameters

When statistical analyses are reported, confirm that the following items are present in the relevant location (e.g. figure legend, table legend, main text, or Methods section).

n/a	Confirmed	
☐	☒	The exact sample size (n) for each experimental group/condition, given as a discrete number and unit of measurement
☐	☒	An indication of whether measurements were taken from distinct samples or whether the same sample was measured repeatedly
☐	☒	The statistical test(s) used AND whether they are one- or two-sided<br>*Only common tests should be described solely by name; describe more complex techniques in the Methods section.*
☐	☒	A description of all covariates tested
☐	☒	A description of any assumptions or corrections, such as tests of normality and adjustment for multiple comparisons
☐	☒	A full description of the statistics including central tendency (e.g. means) or other basic estimates (e.g. regression coefficient) AND variation (e.g. standard deviation) or associated estimates of uncertainty (e.g. confidence intervals)
☐	☒	For null hypothesis testing, the test statistic (e.g. F, t, r) with confidence intervals, effect sizes, degrees of freedom and P value noted<br>*Give P values as exact values whenever suitable.*
☐	☒	For Bayesian analysis, information on the choice of priors and Markov chain Monte Carlo settings
☐	☒	For hierarchical and complex designs, identification of the appropriate level for tests and full reporting of outcomes
☐	☒	Estimates of effect sizes (e.g. Cohen's d, Pearson's r), indicating how they were calculated
☐	☒	Clearly defined error bars<br>*State explicitly what error bars represent (e.g. SD, SE, CI)*

Our web collection on statistics for biologists may be useful.

Software and code

Policy information about availability of computer code

Data collection	Image Lab, BD FACSDiva software
Data analysis	Graphpad Prism 5, Flowjo

For manuscripts utilizing custom algorithms or software that are central to the research but not yet described in published literature, software must be made available to editors/reviewers upon request. We strongly encourage code deposition in a community repository (e.g. GitHub). See the Nature Research guidelines for submitting code & software for further information.

Data

Policy information about availability of data

All manuscripts must include a data availability statement. This statement should provide the following information, where applicable:

- Accession codes, unique identifiers, or web links for publicly available datasets
- A list of figures that have associated raw data
- A description of any restrictions on data availability

Data are available from the corresponding authors upon reasonable request.

Field-specific reporting

Please select the best fit for your research. If you are not sure, read the appropriate sections before making your selection.

☒ Life sciences ☐ Behavioural & social sciences ☐ Ecological, evolutionary & environmental sciences

For a reference copy of the document with all sections, see nature.com/authors/policies/ReportingSummary-flat.pdf

Life sciences study design

All studies must disclose on these points even when the disclosure is negative.

Sample size	The sample size for each experiment, n, is included in the results section and the associated figure legend.
Data exclusions	No data were excluded from the analyses.
Replication	Data are representatives of three independent experiments with similar results and plotted as mean ± SD
Randomization	Mice were random allocated into experimental group
Blinding	The investigators were blinded to group allocation during data collection and analysis.

Reporting for specific materials, systems and methods

Materials & experimental systems

n/a	Involved in the study	
☒	☐	Unique biological materials
☐	☒	Antibodies
☒	☐	Eukaryotic cell lines
☒	☐	Palaeontology
☐	☒	Animals and other organisms
☒	☐	Human research participants

Methods

n/a	Involved in the study	
☒	☐	ChIP-seq
☐	☒	Flow cytometry
☒	☐	MRI-based neuroimaging

Antibodies

Antibodies used

In vivo depletion: anti-mPDCA-1 IgG, clone JF05-1C2.4.1 Miltenyi Biotec Cat#: 130-092-550
ELISA: Anti-mouse IL-1β purified eBioscience Cat#: 14-7012-85
ELISA: Anti-mouse IL-1β biotin eBioscience Cat#: 13-7112-85
ELISA: Anti-mouse IL-6 purified eBioscience Cat#: 14-7061-85
ELISA: Anti-mouse IL-6 biotin eBioscience Cat#: 13-7062-85
ELISA: Anti-mouse IFN-γ purified eBioscience Cat#: 14-7312-85
ELISA: Anti-mouse IFN-γ biotin eBioscience Cat#: 13-7311-81
Western: IL-1β Cell Signaling Technology Cat#: 12426S
Western: Casp1 (p20) Santa Cruz Cat#: SC-398715
Western: Casp1 (p10) Santa Cruz Cat#: SC-514
Western: β-actin Cell Signaling Technology Cat#: 12262S

Validation

All antibodies are commercial available and validated.

Animals and other organisms

Policy information about studies involving animals; ARRIVE guidelines recommended for reporting animal research

Laboratory animals

Mouse: WT: C57BL/6J The Jackson Laboratory JAX: 000664
Mouse: Il1r1-/- The Jackson Laboratory JAX: 003245
Mouse: Aim2-/- The Jackson Laboratory JAX: 013144
Mouse: Nlrp3-/- The Jackson Laboratory JAX: 021302
Mouse: Casp1-/- The Jackson Laboratory JAX: 016621
Mouse: Myd88-/- The Jackson Laboratory JAX: 009088

	Mouse: Irf3-/-Irf7-/- Dr. Kate Fitzgerald Dr. Tadatsugo Taniguchi<br>Mouse: Traf3flox/flox Dr. Shao-Cong Sun<br>Mouse: CD11c-Cre The Jackson Laboratory JAX: 008068<br>Mouse: Zbtb46-DTR The Jackson Laboratory JAX: 019506<br>Mouse: Stat1-/- The Jackson Laboratory JAX: 012606<br>Mouse: Sting-/- The Jackson Laboratory JAX: 017537<br>Mouse: Mavs-/- The Jackson Laboratory JAX: 008634<br>8 weeks old female mice were used for experiments.
Wild animals	The study did not involve wild animals.
Field-collected samples	All mouse-related procedures were performed according to experimental protocols approved by the Animal Care and Welfare Committee at Houston Methodist Research Institute and in accordance with NIH–approved animal study protocol LMVR-11E.

Flow Cytometry

Plots

Confirm that:

☒ The axis labels state the marker and fluorochrome used (e.g. CD4-FITC).

☒ The axis scales are clearly visible. Include numbers along axes only for bottom left plot of group (a 'group' is an analysis of identical markers).

☒ All plots are contour plots with outliers or pseudocolor plots.

☒ A numerical value for number of cells or percentage (with statistics) is provided.

Methodology

Sample preparation	Sample preparation are included in figure legend and methods.
Instrument	BD Aria II
Software	BD FACSDiva
Cell population abundance	More than 20,000 cells after gating were collected for data analysis.
Gating strategy	Gating strategies used for FACS data are provided in the supplementary information

☒ Tick this box to confirm that a figure exemplifying the gating strategy is provided in the Supplementary Information.
